# A Biomathematical Model of Human Erythropoiesis under Erythropoietin and Chemotherapy Administration

**DOI:** 10.1371/journal.pone.0065630

**Published:** 2013-06-06

**Authors:** Sibylle Schirm, Christoph Engel, Markus Loeffler, Markus Scholz

**Affiliations:** 1 Institute for Medical Informatics, Statistics and Epidemiology, University of Leipzig, Leipzig, Germany; 2 LIFE Research Center of Civilization Diseases, University of Leipzig, Leipzig, Germany; University of Medicine and Dentistry of New Jersey, United States of America

## Abstract

Anaemia is a common haematologic side effect of dose-dense multi-cycle cytotoxic polychemotherapy requiring erythrocyte transfusions or erythropoietin (EPO) administration. To simulate the effectiveness of different EPO application schedules, we performed both modelling of erythropoiesis under chemotherapy and pharmacokinetic and dynamic modelling of EPO applications in the framework of a single comprehensive biomathematical model. For this purpose, a cell kinetic model of bone marrow erythropoiesis was developed that is based on a set of differential compartment equations describing proliferation and maturation of erythropoietic cell stages. The system is regulated by several feedback loops comprising those mediated by EPO. We added a model of EPO absorption after injection at different sites and a pharmacokinetic model of EPO derivatives to account for the effects of external EPO applications. Chemotherapy is modelled by a transient depletion of bone marrow cell stages. Unknown model parameters were determined by fitting the predictions of the model to data sets of circulating erythrocytes, haemoglobin, haematocrit, percentage of reticulocytes or EPO serum concentrations derived from the literature or cooperating clinical study groups. Parameter fittings resulted in a good agreement of model and data. Depending on site of injection and derivative (Alfa, Beta, Delta, Darbepoetin), nine groups of EPO applications were distinguished differing in either absorption kinetics or pharmacokinetics. Finally, eight different chemotherapy protocols were modelled. The model was validated on the basis of scenarios not used for parameter fitting. Simulations were performed to analyze the impact of EPO applications on the risk of anaemia during chemotherapy. We conclude that we established a model of erythropoiesis under chemotherapy that explains a large set of time series data under EPO and chemotherapy applications. It allows predictions regarding yet untested EPO schedules. Prospective clinical studies are needed to validate model predictions and to explore the feasibility and effectiveness of the proposed schedules.

## Introduction

Anaemia is a common side-effect of dose-dense multi-cycle polychemotherapies which may result in delays of treatment or reduction of chemotherapy dose [1]. The temporal dynamics of erythrocyte concentrations in the peripheral blood differ from those of leukocytes or thrombocytes. While the latter two cell types are usually characterised by strong oscillations during chemotherapy cycles, erythrocyte counts show only mild oscillations but a progressive decline during the course of the therapy due to the longer half-life of erythrocytes. Anaemia is routinely treated by erythrocyte transfusions or erythropoietin (EPO) administration.

We demonstrated in the past that mathematical models of granulopoiesis and thrombopoiesis can be used to identify optimal chemotherapy and growth factor schedules [2]–[5]. In the present paper, we aim to complete our models by the erythropoietic lineage under chemotherapy and EPO applications. The optimal usage of EPO prophylaxis is largely unknown, due to the complex interaction of chemotherapy toxicity and stimulation of bone marrow by EPO. Therefore, we aim at constructing a model which allows predictions regarding the outcome of EPO prophylaxis in dependence on the EPO derivative used and the chemotherapy applied.

We present an ordinary differential equations model which combines four model concepts developed in different contexts: First, a cell kinetic model describing the development of mature erythrocytes from haematopoietic stem cells and its regulation by endogenous EPO was established by our group [6]–[10]. Second, the cytotoxic effect of chemotherapy is introduced by a model which we successfully applied to granulopoiesis and thrombopoiesis [2], [5], [11], [12]. Third, in order to model external administrations of EPO, we adopted a pharmacokinetic (PK) model of EPO proposed by Krzyzanski & Wyska [13] and combined it with our cell-kinetic model. Finally, different EPO injection sites are considered by attaching an EPO injection model which was originally developed for sheeps [14].

Rather than constructing a model from the scratch, it is our major intention to built it on these established structures. Hence, we adopt their assumptions, equations and parameters as far as possible. This approach takes into account that the developement of a comprehensive model of erythropoiesis under chemotherapy and EPO application in a single effort is difficult due to the complexity of the underlying biological processes. Our paper contributes to a second step of modelling by combining submodels which address single issues of the overall process of interest. However, since all submodels were developed on a limited data base and disjoint biological scenarios, a few adaptation are required to combine them. In the present paper, we describe these assumptions and modifications in detail. A complete set of equations can be found in file S1.

Unknown parameters of the model are estimated by optimising the agreement of model predictions with data of a large variety of published clinical data sets comprising four different EPO derivatives (EPO Alfa, Beta, Delta, Darbepoetin Alfa). These scenarios are accompanied by modelling clinical data obtained from randomised clinical chemotherapy trials of cooperating national study groups. We also validate the model by predicting data not used for parameter fitting and present some model predictions of yet untested combinations of chemotherapy and EPO.

## Methods

### Ethics Statement

Haemoglobin data were taken from published studies of the German High Grade Non-Hodgkin-Lymphoma Study Group [15]–[18] or the German Hodgkin's lymphoma study group [19], [20]. These studies were conducted in accordance with the Declaration of Helsinki. Corresponding protocols were approved by the ethics review committee of each participating center. Patients gave written consent for participation and subsequent analyses of data. A complete list of study centers and corresponding ethics review committees can be found in the appendices of the above mentioned study publications.

### Structure of the Model

**Figure 1:**
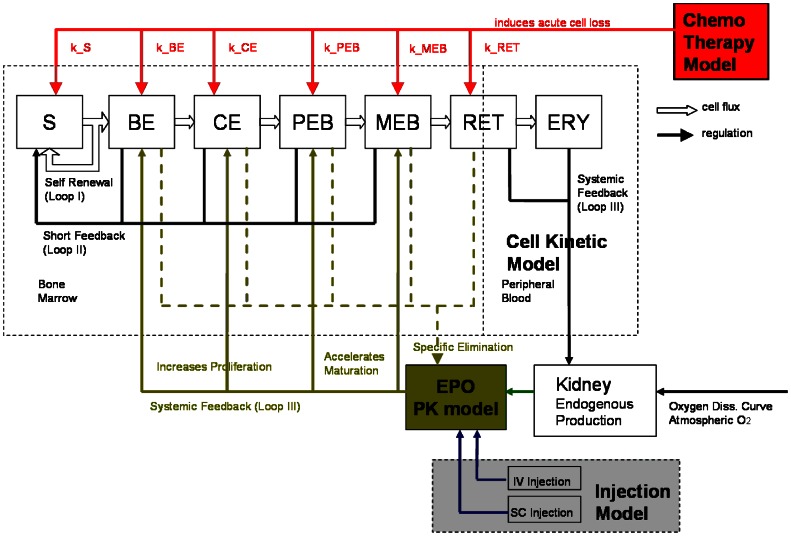
Structure of the model of erythropoiesis under chemotherapy and EPO application. We combined four independently developed models (cell kinetic, pharmacokinetic, injection, chemotherapy) into one comprehensive model. Model compartments are presented in boxes (S = stem cells, BE = burst forming units - erythroid, CE = colony forming units - erythroid, PEB = proliferating erythrocytic blasts, MEB = maturing erythrocytic blasts, RET = reticulocytes, ERY = erythrocytes, EPO = erythropoietin). Several regulatory feedback loops are implemented. The most important one is mediated by EPO which is produced endogenously and can also be applied externally. Chemotherapy is modelled by a transient depletion of cells.

We constructed a model of human erythropoiesis under chemotherapy and EPO applications by combining a cell kinetic model of bone marrow erythropoiesis [6]–[10], a pharmacokinetic model of EPO application [13], an absorption model of different modes of EPO injection into subcutaneous tissue [14] and a model of chemotherapy action originally developed for granulopoiesis and thrombopoiesis [5], [11], [12]. In the following, we briefly sketch the structure of these models and describe modifications necessary for combining them. A schematic representation comprising all parts of the model is shown in figure 1.

#### Cell Kinetic Model

An ordinary differential equations model of bone marrow erythropoiesis was developed in the past (see [6]–[9], [21]). We briefly sketch this model for self-consistancy. The model consists of concatenated cell compartments describing the dynamics of the following bone marrow cell stages of erythropoiesis: haematopoietic stem cells (S), burst forming units - erythroid (BE), colony forming units - erythroid (CE), proliferating erythroblasts (PEB), maturing erythroblasts (MEB), reticulocytes (RET) and mature erythrocytes (ERY). Differentiation of cells is modelled by fluxes from one compartment to the next stage. Proliferation is modelled by amplification in the corresponding compartment (see [21]). Compartment sizes are modelled by balance equations of the form
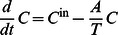
where 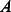 is the amplification, 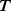 the transit time, 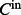 is the influx from the previous compartment, and 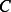 the content of the compartment. The stem cell compartment has a different structure: Here, the percentage of self-renewing cell divisions and the percentage of proliferating cells is regulated by the bone marrow cell compartments (details see section A.2 in file S1). The stem cell compartment is identical to those used in other lineage models of our group [5], [11], [12].

Maturation is modelled by a set of concatenated first order transitions of the form
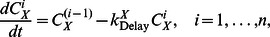
where 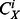 is the content of the 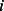-th subcompartment of 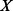, 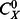 is the influx from the previous compartment and 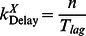 is the delay parameter with delay time 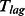. In [11] it has been shown that this results in Gamma-distributed delay times with expectation 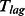 and variance 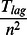. Later, this principle is not only applied to model maturation but also to delay chemotherapy action and (delayed) absorption of EPO injections.

The differential equations system is regulated by several feedback loops. Loop 1 and 2 are feedbacks regarding the stem cell self-renewal and proliferation [9], [22]. Loop 3 is mediated by the cytokine EPO which increases amplification (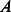) or shortens maturation time (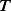) in all bone marrow compartments except for the stem cells [7]. Endogenous EPO production is regulated by oxygen saturation. For a brief explanation of the model of endogenous EPO regulation see the file S1.

Quantities 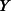 that depend on EPO concentration are typically regulated by so called Z-functions. The Z-function is a monotone function with minimum 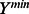 and maximum 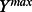. It reads as follows
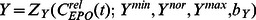
(1)

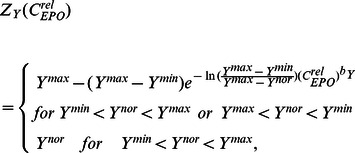
(2)where 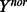 is the steady state value, 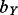 is the sensitivity of 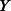 under stimulation and 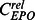 is the EPO concentration relative to steady-state (See also [9], p. 69.)

A further model assumption is that in steady state most erythrocytes die dependent on age (i.e. similar to a first-in-first-out kinetic), but under stimulation, erythrocyte degradation occurs more randomly (i.e. exponential decay) [21]. Finally, iron metabolism is not explicitly modelled and it is assumed that iron supply is not a limiting factor. A complete set of model equations and parameters can be found in the file S1.

This model of bone marrow erythropoiesis was established on the basis of several experimental results for example on irradiation, bleeding, hypoxia and hypertransfusion in mice [9]. But so far, neither applications of EPO nor chemotherapy were considered. Due to its successes in explaining the above mentioned scenarios, we adopt the assumptions and equations of this cell kinetic model. They serve as a backbone of our comprehensive model in the sense that all other parts, i.e. pharmacokinetics and -dynamics of EPO and chemotherapy injections, are attached to this structure.

#### Pharmacokinetic Model of Erythropoietin

A pharmacokinetic model of EPO was developed by Krzyzanski & Wyska [13] to explain dynamics of EPO serum concentrations in healthy volunteers after intravenous injections. Neither chemotherapy patients nor subcutaneous applications of different EPO derivatives were considered there. In brief, the model consists of three compartments: EPO in central serum (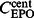), EPO protein binding (peripheral compartment (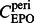) and EPO bound to receptors (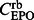). EPO is eliminated at the rate 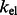 from the central compartment. There is a first order exchange between the peripheral and the central compartment with the rates 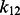 and 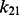 respectively. EPO in central compartment can bind to free EPO receptors 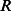 at the rate 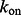 forming the drug-receptor complex 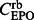. The complex dissociates with rate 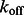. Alternatively, EPO is internalised with rate 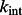. The parameter 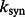 describes the receptor synthesis rate and 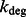 is the degradation rate for EPO receptors [13].

Coupling this pharmacokinetic model with our cell kinetic model is achieved by three mechanisms:

**Model assumption 1:** There is an additional influx of endogenously produced EPO into the central compartment. Endogenous EPO is produced in accordance to the cell kinetic model.

**Model assumption 2:** The production of EPO receptors depends on the bone marrow cellularity, i.e. the compartments BE, CE, PEB, MEB, RET of the cell kinetic model.

**Model assumption 3:** Internalised EPO serves as the argument of all Z-functions of the cell kinetic model representing EPO regulations.

**Figure 2:**
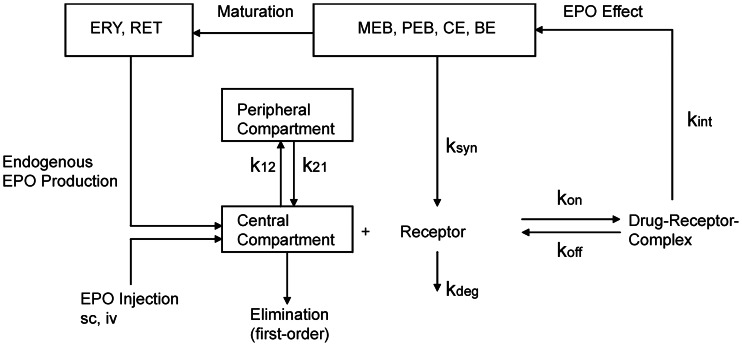
Pharmacokinetic model of EPO. Boxes represent compartments. Arrows represent actions or flows. The pharmacokinetic model of erythropoietin was adapted from [13].

All three assumptions are natural extensions of the model. A schematic illustration is shown in figure 2. The first assumption translates in the following (adapted) equation of the central compartment:
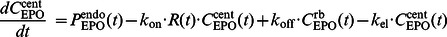


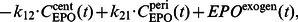
(3)where 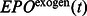 is a function of external EPO applications specified later. The function 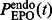 of endogenous EPO production is defined as follows:
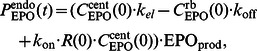
(4)where 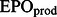 equals one in steady state, so that equation 3 equals zero. 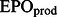 depends on the oxygen partial pressure and the number of circulating red blood cells (details see section A.2 in the file S1 or [7]–[9]).

Dynamics of the peripheral compartment and the EPO-receptor complex are described by the following equations [13]:
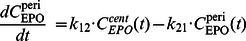


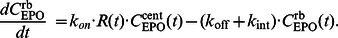



The specific elimination of EPO depends on the number of EPO receptors. According to assumption 2, we assume that the dynamics of EPO-receptors 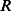 can be calculated on the basis of the bone marrow content of the cell kinetic model, namely the compartments 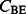, 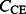, 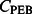, 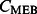, and 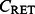. The different receptor densities of these cell states are modelled by weighting factors 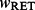, 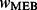, 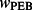, 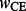, and 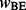. The highest weighting factor is applied for CFU-E due to the highest number of EPO receptors [23]. We further assume that the density of EPO receptors declines with further maturation. Hence 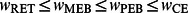 and 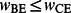. Mature erythrocytes are assumed to show no specific binding of erythropoietin [23], [24]. Hence
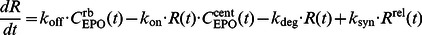
(5)where 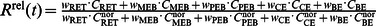 is the number of EPO receptors relative to steady-state.

We now derive the initial values of the differential equations. At first, we set.
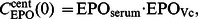
where 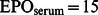 IU/l is the basic level of endogenous EPO, and 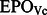 denotes the distribution volume of EPO [13]. In consequence, steady-state concentration of EPO in central compartment is equal to the observed (mean) serum concentration.

Initial values of the other equations are also derived from steady-state conditions:
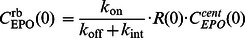


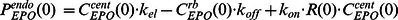


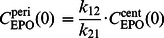



According to [13], we set 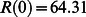 which implies that the parameter 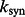 is not free because from equation 5 it follows that 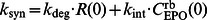.

The coupling of the pharmacokinetic model and the cell kinetic model is completed by using the relative internalised EPO (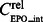) as argument of the Z-functions (equation 2) of all parameters regulated by EPO, namely amplifications (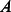) and transition times (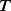)
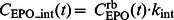


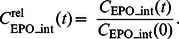



#### Model of EPO Injections

External EPO applications are modelled by an additional influx 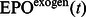 into the central compartment of the EPO pharmacokinetic model (equation 3). Different modes of EPO application were tested in clinical practice resulting in different absorption kinetics. While intravenous injections can be simply modelled by pulse functions 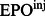 (see equation 6 below), subcutaneous injections are characterised by delayed and incomplete absorption.

**Figure 3:**
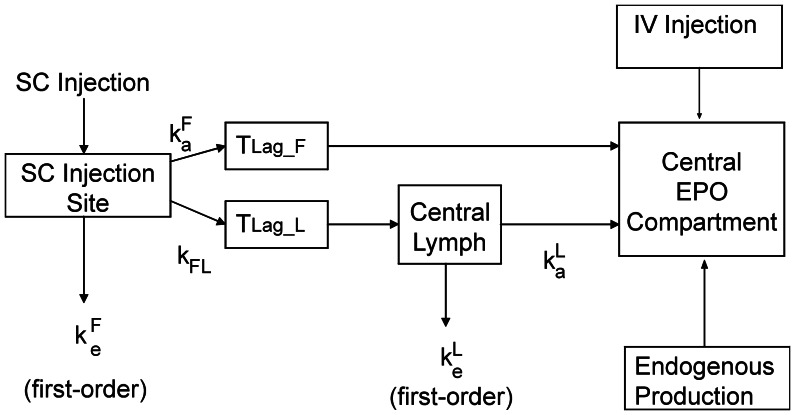
Structure of the EPO injection model with two ways of EPO absorption. The model is in analogy to [14]. Boxes denote the compartments, arrows represent EPO fluxes between the compartments.

Kota *et al.* [14] proposed a model of Darbepoetin absorption in sheeps explaining absorption kinetics of subcutaneous EPO applications at different sites. Different models were considered for different sites of application. We adapted model proposal B of Kota *et al.* for our purposes. EPO absorption is determined by two processes, directly to the bloodstream or indirectly via the lymphatic system. Both processes are delayed. There is also a loss of EPO at the injection site and in the lymphatic system. In comparison to model proposal B of Kota *et al.* we made the following simplifications (see also figure 3):

**Model assumption 4:** First order absorptions of EPO into central compartment or lymphatic system are neglected, i.e. we only assume delayed absorptions.

**Model assumption 5:** Different EPO derivatives and injection sites are modelled by the same equations but different parameters.

This results in the following equations. The general EPO injection function 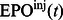 can be written as a sum of pulse functions
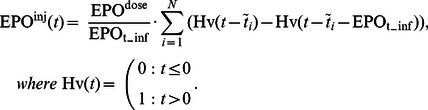
(6)is the Heaviside-function, 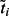 are time points at which EPO at dose 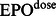 is administered. The duration of injection (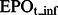) is set to five minutes. The applied dose is normalised to this injection time by the factor 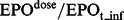. 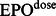 is given by the administered dose in IU/kg. EPO dynamics at the injection site (subcutaneous tissue) 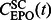 are then described by:
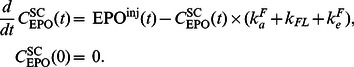
(7)where 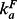 is the efflux constant towards the central compartment, 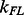 is the efflux constant towards the lymphatic system and 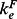 represents a first order loss term of EPO resulting in a reduced bioavailability. The direct efflux from subcutaneous compartment into peripheral blood is delayed by four delay compartments, i.e.
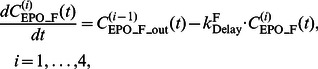
(8)with the settings



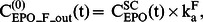
and



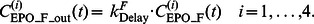




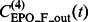
 finally enters the central EPO compartment of the PK model (equation 3, see also equation 12). Analogously, the lymphatic absorption is modelled by a delay function with four compartments:
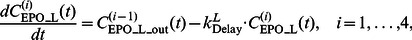
(9)with the settings



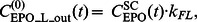
and



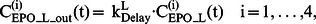
where 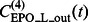 enters the central lymph compartment. Thus, EPO dynamics in the lymphatic compartment 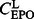 are given by



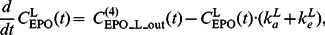
(10)

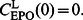
(11)where 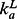 is the absorption constant of the transition between the lymphatic and the central compartment. The constant 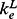 represents another loss term resulting in further reduction of the bioavailability via this route of absorption. Summarising the effluxes of the direct and the lymphatic way of absorption yields
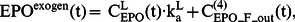
(12)for subcutaneously administered EPO and
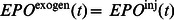
(13)for intravenous injections. Due to differences in the lymphatic flow at different anatomical regions [14], [25], [26], one cannot assume that the same parameters of the injection model are valid for all injection sites considered. In contrast to [14] we do not assume differences in the structure of the model in dependence on the injection site. Rather than this, EPO derivatives (Alfa, Beta, Delta, Darbepoetin Alfa) and injection sites used in our clinical data sets were modelled by different parameter sets of the same injection model (assumption 5). For this purpose, we split available injection modi into 9 groups for which different parameter sets are assumed (see later).

#### Chemotherapy Model

We recently proposed a model of cytotoxic chemotherapy action on the granulopoietic system [5], [11], [12]. This concept is translated to our erythropoiesis model. It is based on the following assumptions:

- Chemotherapy results in a reversible and transient depletion of the compartments S, BE, CE, PEB, MEB, and RET. None of the other compartments or model parameters are affected by chemotherapy applications [11].
- The damaging effect is delayed, modelled by four subcompartments in analogy to equations 8, 9 [51]. This implies that the damaging effect is zero at time of injection and maximal at a later time point which can be specified by the delay parameter.
- The toxic effect is specific for the cell stages and for different drugs or drug combinations. It is quantified by corresponding sets of *toxicity parameters* [2].
- The toxicity is higher for the first administration of a chemotherapeutic drug (first cycle effect) which is modelled by a factor 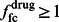 multiplied to all toxicity parameters for the first therapy cycle [11].
- Different cytotoxic drugs damage independently of each other [11]. This allows adding toxicity functions of chemotherapeutic drugs applied in combinations.

These assumptions are extensively discussed in [5], [11], [12]. We now present the corresponding equations. The infusion of chemotherapeutic drugs is again modelled by Heaviside functions
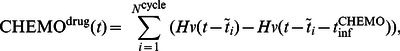
(14)where 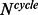 is the number of chemotherapy cycles and 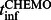 is the duration of chemotherapy application. The delayed effect of the drugs is again modelled by four compartments
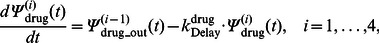
(15)with



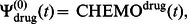


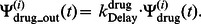



The output-function 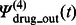 is multiplied by the toxicity parameters of the single compartments 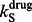, 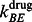, 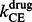, 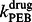, 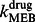, and 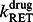 respectively. Since both, our former model of granulopoiesis and the present model of erythropoiesis use the same stem cell compartment, the parameters 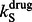, 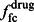, and 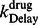 were taken from the granulopoiesis model described in [5], for consistency. All other toxicity parameters are unknown and were estimated from clinical data (see below). If multiple cytotoxic drugs are applied, corresponding toxicity functions are added resulting in an overall toxic effect 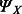 which is cell stage and chemotherapy specific. In complete analogy to our granulopoiesis model, the effect of chemotherapy is included into the balance equations of the bone marrow cell compartments by a first-order loss term. Hence, the schematic compartment equation 1 has the form
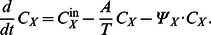



### Model Calibration, Parameter Estimation and Validation

#### Numerical methods for simulation

Implementation and simulation of the model were performed using MATLAB 7.5.0.342 (R2007b) with SIMULINK toolbox (The MathWorks Inc., Natick, MA, USA). Numerical integrations of the equation system were performed using the variable step solver from Adams and Bashford included in the SIMULINK toolbox (ode113).

#### Estimation of parameters

Data on bone marrow erythropoiesis are hardly available for humans. Consequently, one can find only a few hints regarding values of certain model parameters in the literature. The majority of model parameters must be estimated indirectly via fitting the predictions of the model to clinical data after various therapeutic interventions. This applies for parameters of all submodels: the cell kinetic model, the PK model, the injection model and the chemotherapy model.

Even if parameter estimates of the submodels were provided in general, it was necessary to adapt these parameters in view of the greater data base to be explained with our model. For example, the injection model was developed for sheeps and must be re-parametrised for humans. The original PK model [13] was developed for intravenous EPO-Alfa injections but not for (subcutaneous) injections of other EPO derivatives. The cell kinetic model never considered external EPO applications so far. This implies that new pharmacodynamic parameters must be estimated.

The effects of chemotherapy on this cell kinetic model were also not studied in the former publications. Modelling different chemotherapy regimens is of particular concern since it requires estimation of corresponding toxicity parameters. In general, these parameters describe the toxicity of a drug combination, since cytotoxic drugs are usually applied as polychemotherapy including several drugs at the same time. However, by comparing different chemotherapies, it is sometimes possible to estimate the toxicity of single drugs.

To reduce the number of parameters to be fitted, we used the following approach:

- We made simple model assumptions to reduce the complexity of equations, and with it, the number of unknown parameters.
- Parameters for which experimental data are available were taken from the literature, either directly or by defining plausible ranges.
- Parameter values of the cell kinetic model and the chemotherapy model which were determined by previous modelling works were kept constant. This applies for parameters of the stem cell regulation and chemotherapy toxicity.
- Different EPO derivatives and injection sites were grouped according to expected similarities regarding absorption kinetics. Parameters of the injection model were assumed to be constant within these groups but may differ between groups. Nine such groups were identified (see below). PK parameters were assumed to be constant for endogenous EPO and the derivatives Alfa, Beta, Delta but are assumed to be different for Darbepoetin.

Parameters of our model were determined by a stepwise fitting procedure. First, pharmacokinetic and -dynamic parameters (PD) of EPO and parameters of the cell kinetic model were estimated by comparing the predictions of the model with available time series data after application of EPO into healthy volunteers. Then, data of different chemotherapy regimens are used to identify corresponding toxicity parameters. Parameters are kept constant in the following fitting process. Fitting is achieved by optimising the agreement of model and data via the condition.
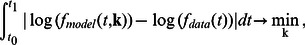
(16)where 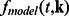 is the solution of the differential equation system at time 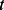 for the parameter set 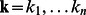. Here, 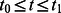 describe the range of time points for which data are available. The function 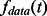 represents the interpolated curve of patients medians for a given data set. We used the logarithm of data due to log-normally distributed cell counts and cytokine concentrations. The left hand side of equation (16) is referred as the fitness function in the following. The simultaneous fit of more than one data set is realised by adding corresponding fitness functions.

We used (1+3)-evolutionary-strategies with self-adapting mutation step size to find optimal parameter sets as good as possible [27], [28]. Evolutionary strategies are non-deterministic optimisation algorithms adopting principles of evolution, namely mutation, realisation and survival of the fittest. (1+3) is an evolution strategy with one possibly immortal parent having three children in each generation (see [27], [28] for further details). The weighting factors of equation 5 were fitted with the restriction 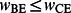, 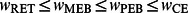 (see [23]). For the toxicity parameters we claim that higher doses correspond to the same or larger toxicity parameters. These restrictions are fulfilled by rejecting corresponding violations during the fitting process.

A complete parameter list for our model is provided in the file S1. We also performed an extensive sensitivity analysis in order to determine the identifiability of all model parameters. This is achieved by estimating the deterioration in fitness after changing the parameters. Results can also be found in the file S1.

#### Data Sets

Available data sets comprised time course data of haemoglobin, haematocrit, reticulocyte counts, percentage of reticulocytes, red blood cell counts or serum concentration of EPO after single or multiple applications of four different EPO derivatives (Alfa, Beta, Delta, Darbepoetin Alfa) to healthy volunteers at a large variety of injection sites. These data were taken from the literature. Automated tools were used to extract the data from the figures as precisely as possible (software “ycasd”, developed by A. Gross, publication in progress [29]). Additionally, time series data of haemoglobin in patients treated with chemotherapy and with or without EPO support were available. A total of 12 chemotherapies were considered. For most of them we have access to raw patient data due to collaborations with the *German High Grade Non-Hodgkin's-Lymphoma Study Group* and the *German Hodgkin's Lymphoma Study Group*. For our purposes, we censored patient data at the time of first erythrocyte transfusion. Patient data of the same chemotherapy arm, cycle number and cycle day were pooled in order to obtain median time courses under the different therapies. Data sets of patients with anaemia due to kidney failure were intentionally not considered so far since this clinical situation requires other model assumptions. An overview of all available data sets is presented in table 1.

**Table 1:** Available data sets.

EPO	chemotherapy	disease	reference
Alfa	none	none	[33]
Alfa	none	none	[60]
Alfa	none	none	[61]
Alfa	none	none	[62]
Alfa	none	none	[54]
Alfa	none	none	[31]
Alfa	none	none	[63]
Alfa	none	none	[64]
Beta	none	none	[55]
Beta	none	none	[32]
Beta	none	none	[56]
Beta	none	none	[26]
Beta	none	none	[25]
Delta	none	none	[27]
Darbepoetin Alfa	none	none	[58]
Alfa	E-T-C	breast cancer	[65]
Darbepoetin Alfa	Platinum,	Small-Cell	[59]
	Etoposide-21	Lung Cancer	
none	Platinum,	Small-Cell	[59]
	Etoposide-21	Lung Cancer	
none	CHOP-21*	NHL	[15], [16]
none	CHOEP-21*	NHL	[15], [16]
none	BEACOPP-21*	HD	[19]
none	CHOP-14*	NHL	[15], [16]
none	CHOP-14*	NHL	[18]
none	CHOEP-14*	NHL	[15], [16]
none	high-	NHL	[17]
	CHOEP-21*		
none	BEACOPP-14*	HD	[20]
none	BEACOPP -21	HD	[19]
	escalated*		
none	E-T-C	breast cancer	[65]
none	EC-T	breast cancer	[65]

Upper part: Data sets regarding application of different EPO derivatives in healthy volunteers. Lower part: Chemotherapy data sets, those with access to raw data are indicated with an asterisk. HD = Hodgkin's disease, NHL = aggressive non-Hodgkin lymphoma.

Not all of these scenarios were utilised for parameter fitting. A few scenarios were kept in reserve for model validations (see results). Since our model describes the dynamics of reticulocytes and erythrocytes, a set of transformation formulas are required to compare model results with available dynamics of haemoglobin (HB) or haematocrit (HCT). Baseline HB values showed a large variance between the heterogeneous populations in the data sets. Since relative compartment sizes were modelled, comparison with data can be achieved by normalising values to baseline. We assume proportionality of HB and the numbers of reticulocytes and erythrocytes. Thus,
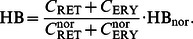
(17)


Analogously, we use the baseline RBC as normalisation factor 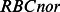 and calculate the changes in this cell count by
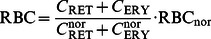
(18)


Percentages of reticulocytes and the haematocrit were determined by
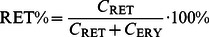


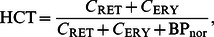


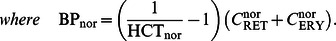
Here, 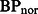 is the amount of plasma which is assumed to be constant for all scenarios. Most of our data sets comprise time series of either percentage of reticulocytes (14 scenarios) or HB (22 scenarios).

## Results

We first study the qualitative behaviour of our model on the basis of our parameter estimates.

### Model Behaviour

**Figure 4:**
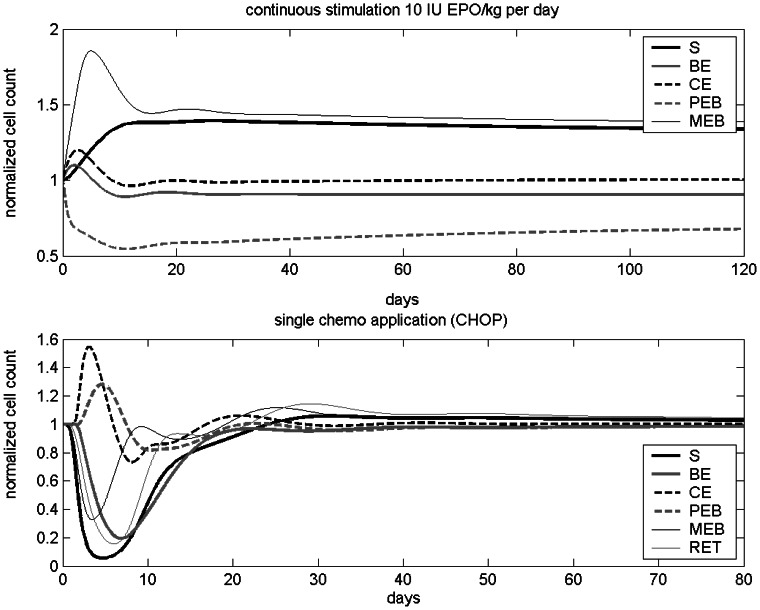
Model dynamics after single perturbations. A) Continuous EPO stimulation. B) Effect of a single cycle of CHOP chemotherapy.

Model dynamics after single perturbations were investigated. A constant influx of 10 IU EPO per day (Alfa, i.v.) results in a new steady state after a damped oscillation (see figure 4). The compartments MEB, RET and ERY are inflated while the compartment PEB is reduced compared to the unperturbed steady state. The compartment S is also inflated due to loop 2. The values of the compartments BE and CE are close to those of the unperturbed steady state.

Next we studied the behaviour of the relative cell counts of all compartments after a single application of CHOP chemotherapy. After a damped oscillation, the system returns to steady state in about 20–40 days (see figure 4).

### Injection Model, Pharmacokinetic Model and Cell Kinetic Model

The injection model, the cell kinetic model, and the pharmacokinetic model were parameterised in one step. Nine different groups of subcutaneous injection modes were distinguished according to available data sets in the literature, namely injections of EPO Alfa into thigh, injections of EPO Alfa into shoulder, injections of EPO Alfa into forearm, injections of EPO Alfa into upper arm or abdomen, injections of EPO Beta into forearm, injections of EPO Beta into arm or abdomen, injections of EPO Beta into thigh, injections of EPO Delta, injections of Darbepoetin Alfa. While the injection parameters differ between groups, the PK parameters are the same for all EPO derivatives and equal to those of endogenous EPO except for Darbepoetin. Parameters of the injection and pharmacokinetic model were determined by fitting the predictions of the model to data of EPO applications into healthy volunteers.

**Figure 5:**
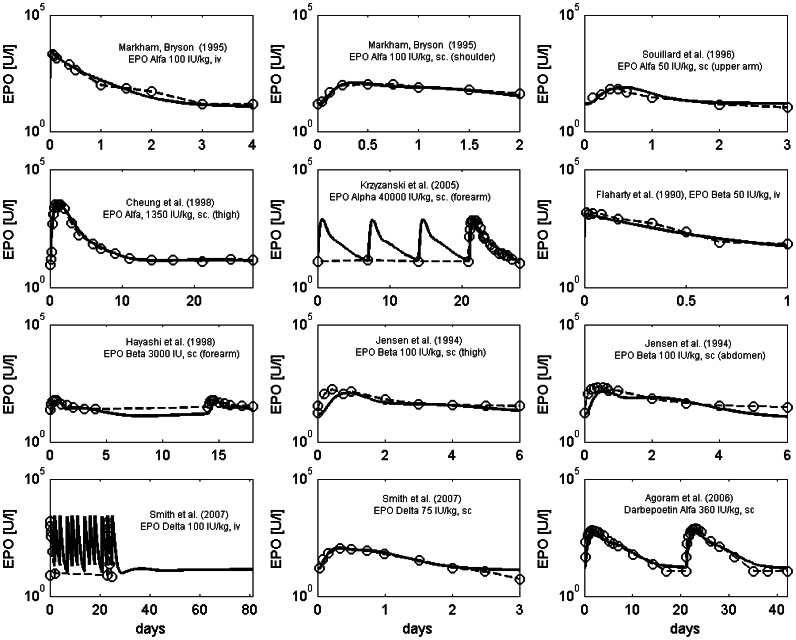
Comparison of model prediction and data of EPO serum concentration for different injection scenarios. The data are taken from the following literature: [13], [26], [31], [33], [54]–[58].

We simulated 42 scenarios differing in EPO derivatives applied, injection sites, doses and schedules and compared the predicted EPO serum concentrations with the data. Parameter estimates of the injection model and the pharmacokinetic model resulted in a good agreement of model predictions and data of EPO serum concentration for almost all scenarios. Examples are shown in figure 5. Results of all scenarios can also be found in the file S1. Parameter estimates of the injection model can be found in the file S1.

**Figure 6:**
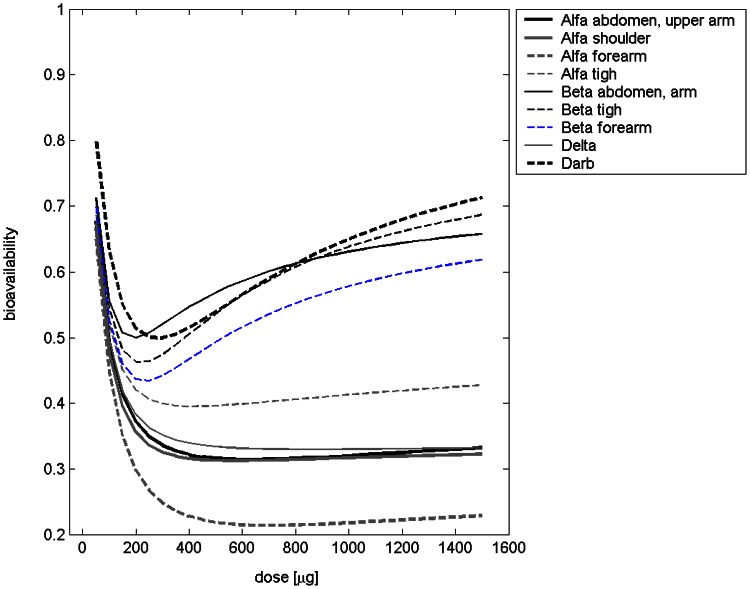
Estimated bioavailability of erythropoietin after different types of injection and in dependence on dose. Here, bioavailability is defined as the ratio of the integrated influxes into the central compartment under subcutaneous and intravenous injection respectively.

To illustrate the differences between derivatives and injection sites, we calculated the bioavailability and the percentage of drug absorbed via the lymphatic system. Here, we define bioavailability as the ratio of integrated influxes into the central compartment under subcutaneous and intravenous injection respectively (figure 6, table 2). Due to linearity of the equations, the lymphatic absorption is independent of the EPO dose applied (table 2). The estimated percentages of lymphatic absorption range from 40.3% for Alfa injected into the thigh to 82.5% for Alfa injected into the shoulder. The estimated bioavailabilities range from 21.4% for Alfa injected into the forearm to 63.9% for Beta injected into the thigh. The scenarios Alfa into shoulder, Alfa into forearm, and Delta showed similar bioavailabilities and importance of lymphatic absorption. If EPO Alfa is applied to thigh, abdomen or upper arm, the importance of lymphatic absorption appears to be smaller and the bioavailability appears to be higher compared to the other modes of EPO Alfa injection considered. For EPO Beta we estimated the highest bioavailability of all first-generation EPO derivatives. The importance of lymphatic absorption is comparable for the different injection sites of EPO Beta.

**Table 2:** Derived quantities based on parameters of the injection model.

		bioavailability	lymphatic absorption
Alfa	abdomen, upper arm [31], [61], [63]	0.314–0.494	0.556
Alfa	shoulder [54]	0.313–0.477	0.825
Alfa	forearm [64]	0.214–0.448	0.799
Alfa	thigh [33]	0.395–0.519	0.403
Beta	abdomen [26]	0.500–0.631	0.716
Beta	thigh [26]	0.462–0.639	0.668
Beta	forearm [56]	0.434–0.579	0.668
Delta	[57]	0.330–0.495	0.808
Darbepoetin	[58], [59]	0.499–0.650	0.633

EPO dose range 100–1000 IU/kg.

Regarding PK parameters, we distinguished between EPO Alfa, EPO Beta, EPO Delta and endogenous EPO on one hand, and, Darbepoetin-Alfa on the other hand. Corresponding sets are valid for all application modes and doses considered. Unspecific elimination of Darbepoetin (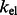) was estimated to be smaller than for the other EPO derivatives. The receptor affinity measured by the dissociation constant (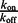) is comparable for both groups. But we estimated that Darbepoetin is internalised more slowly compared to the other derivatives. A complete list of PK parameters can be found in the file S1.

### Cell Kinetic Model

The same pharmacodynamic (PD) parameters were assumed for endogenous EPO and the first generation EPO derivatives (EPO Alfa, EPO Beta, EPO Delta). But for Darbepoetin, PD parameters were allowed to differ which results in two parameter sets for loop 3.

**Figure 7:**
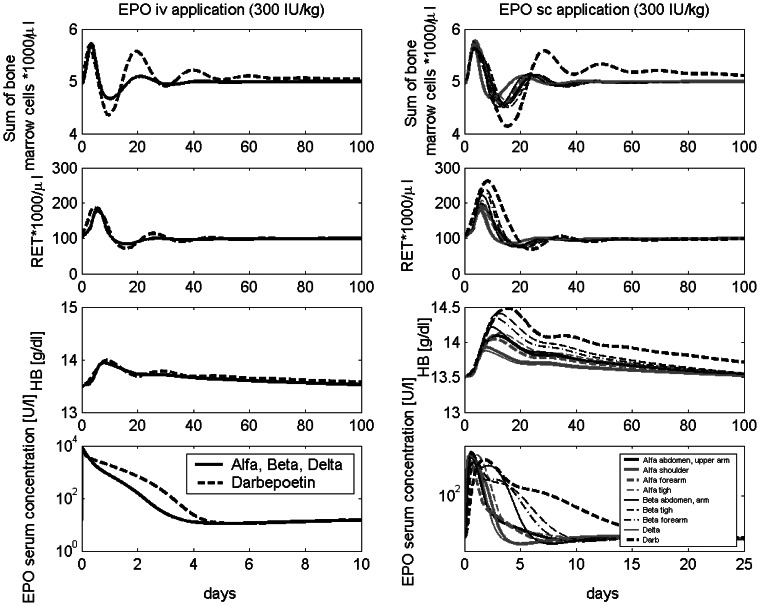
Comparison of injection modes. Comparison of erythrocytes, reticulocytes, HB values, and the sum of the bone marrow cell stages after intravenous (left) and different subcutaneous injection sites (right).

Parameter estimates resulted in a good agreement of model and data for almost all scenarios. Examples are shown in figure 5. Results for all other scenarios can be found in the file S1. To illustrate estimated PD differences between Darbepoetin and the other derivatives, we compared the corresponding Z-functions (see file S1). The regulation of the transition time in the compartments BE, CE, PEB, MEB and the amplification in compartment CE are similar for Darbepoetin and the other EPO derivatives. We estimated that in compartment BE we have less amplification under Darbepoetin compared to the same amount of the other EPO derivatives. The other Z-functions are roughly the same. Pharmacodynamic properties of the drugs are illustrated in figure 7 on the basis of single intravenous or subcutaneous injections. Note that both dynamics also depend on the pharmacokinetic properties of the drugs while the dynamics after subcutaneous injections additionally depend on absorption kinetics at different sites.

### Chemotherapy Model

**Figure 8:**
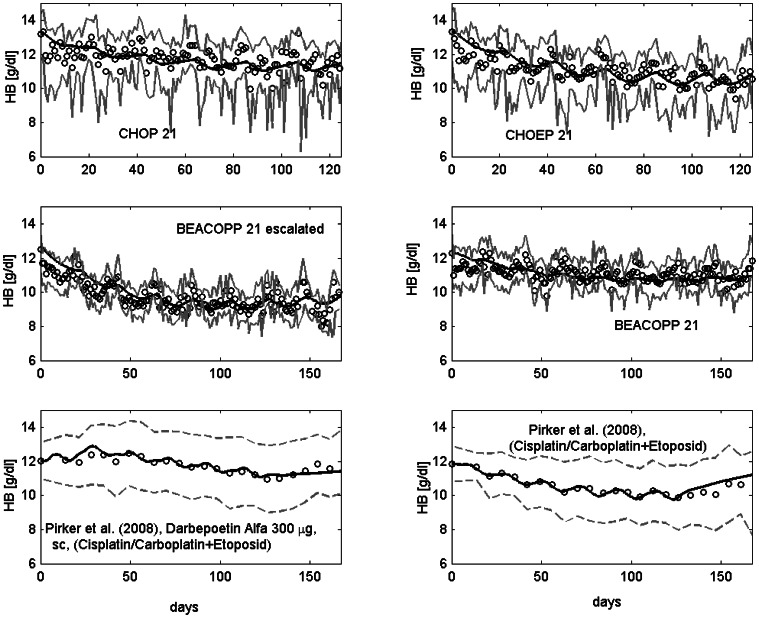
Comparison of model and data for a number of chemotherapy scenarios. Dots represent patient medians (first four) or means (last two), grey solid lines the interquartile range of data, grey dotted lines mean 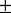 standard deviation. Black solid line is the model prediction.The following chemotherapies are displayed: CHOP, CHOEP, BEACOPP 21 escalated, BEACOPP 21, for which we have access to raw data (studies are described in [15], [16], [19], [20]), and Platinum plus Etoposide with or without Darbepoetin Alfa (data were extracted from the literature [59]).

**Figure 9:**
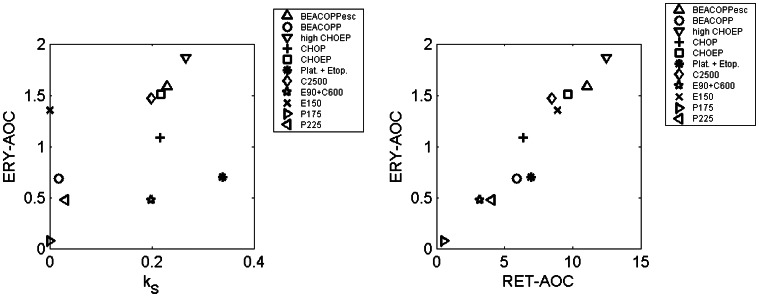
Comparison of toxic effects between different chemotherapies. We present the relations between stem cell toxicities and resulting AOC of reticulocytes and erythrocytes for a single therapy cycle.

After establishing parameter sets of our injection, PK and cell kinetic model, we aim to apply our model to different chemotherapy scenarios. This requires estimation of toxicity parameters which were determined by fitting the predictions of the model to data of patients treated with either BEACOPP-21, BEACOPP-21 escalated, CHOEP-14,21, CHOP-14,21, the sequential chemotherapy schemes EC-T (Epirubicin plus Cyclophosphamide, Paclitaxel), E-T-C (Epirubicin, Cyclophosphamide, Paclitaxel), E-T-C plus EPO, highCHOEP-21, Platinum plus Etoposide or Platinum plus Etoposide with Darbepoetin. For most of these scenarios, only haemoglobin dynamics are available. Toxicity parameter estimates resulted in a good agreement of model predictions and data in the sense that for almost all scenarios and time points, the model curve is in between the 25th and 75th percentile of patients data [5]. Examples are shown in figure 8. All others scenarios can be found in the file S1. A complete list of toxicity parameter estimates can also be found in the file S1. We like to emphasize here, that the parameters of stem cell toxicity, which are most sensitive, were not fitted but retrieved from our granulopoiesis model [5]. To illustrate the overall toxicity of different chemotherapeutic drugs we calculated the area over curve (AOC) for erythrocyte and reticulocyte counts below normal, i.e.
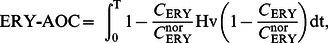


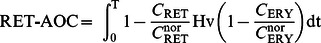
for 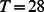 days and a single chemotherapy injection at 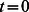. Since in general the stem cell toxicity of a drug or drug combination is the most significant parameter influencing the haematotoxic outcome, we plot the stem cell toxicities against ERY-AOC in figure 9. We also plot RET-AOC against ERY-AOC. As expected, there is a strong correlation of RET-AOC and ERY-AOC. There is also a clear correlation of the estimates of the stem cell toxicities and ERY-AOC. But this correlation is less perfect since damage at later cell stages cannot be neglected in general. For example etoposide (E) is known to be stem cell saving but toxic to later cell stages [30]. The results fit well to our clinical understanding of the chemotherapies: BEACOPP-21 escalated and highCHOEP-21 are the therapies with highest toxicity. CHOP, CHOEP and C2500 have moderate toxicity and application of single drugs except for highly dosed cyclophosphamide (C2500) have relatively low toxicity.

### Validation and Prediction

**Figure 10:**
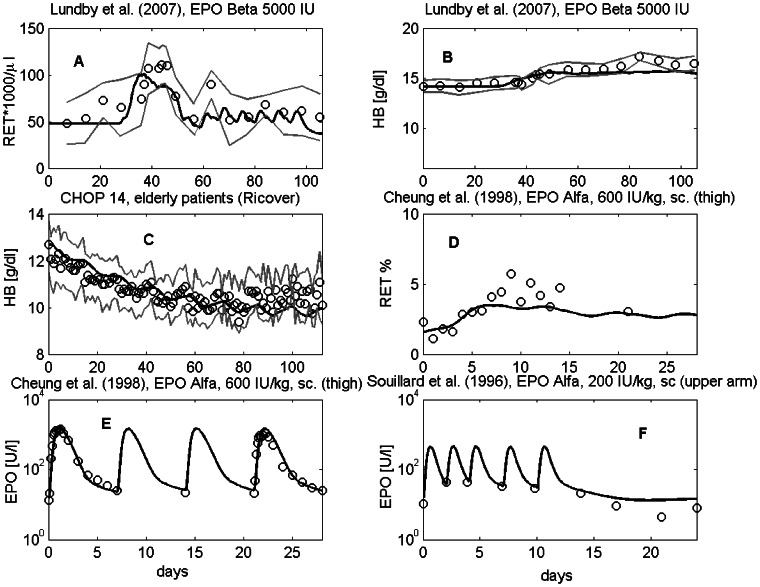
Validation scenarios. Reticulocytes (A) and HB (B) after multiple injections of EPO Beta (data were extracted from [32]): dots represent patient means, grey dotted lines mean 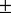 standard deviation. (C) HB under eight cycles of CHOP-14 therapy (we have access to the raw data, the study is described in [18]): dots represent patient medians, grey lines represent interquartile range of patient data. Reticulocytes (%) (D) and EPO serum concentrations (E,F) after multiple EPO Alfa injections at different subcutaneous sites [31], [33]: dots represent patient means. Solid black curve represents model predictions throughout.

A few data sets were not utilised for parameter fitting, but were used for model validation. Data sets can be used for validation if no additional parameters must be fitted, i.e. the chemotherapy toxicities and the injection model parameters are known from a different data set. These data comprise the following scenarios: EPO serum concentration from athletes receiving a single dose of 50 IU/kg or multiple application of a high dose of 200 IU/kg EPO Alfa on day 0, 2, 4, 7, and 10 into the upper arm [31], data of HCT, HB and reticulocyte time courses after multiple injections of 5000 IU EPO Beta over a long time period of 15 weeks [32], as well as one scenario from Cheung *et al.* with weekly injections of 600 IU EPO Alfa into the thigh [33]. Predictions of all scenarios fitted well to the observed clinical data (see figure 10). For validation of our model of chemotherapy, we used time series data of HB under eight cycles of CHOP-14 therapy retrieved from the RICOVER trial [18]. Results of model simulation fit well for the cycles 1–6. But HB values of the last two cycles were slightly underestimated.

**Figure 11:**
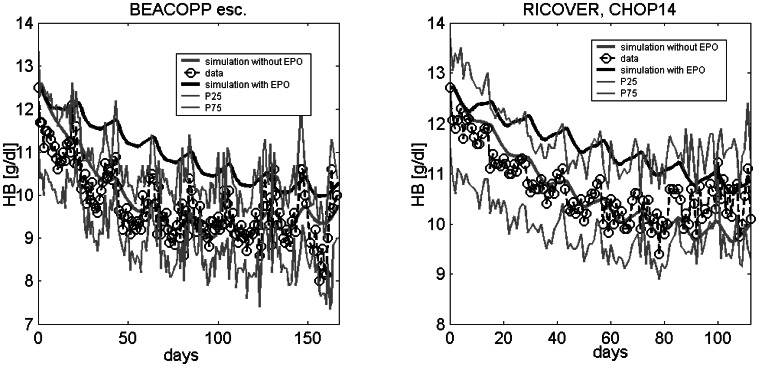
Prediction. Weekly administration of 150 IU/kg Darbepoetin Alfa has the potential to avoid anaemia in patients treated with 8 cycles of BEACOPP-21 escalated (left) or CHOP 14 (right).

After calibration and validation of the model, it can now be used to make clinically relevant predictions. As an example, we study the effect of anaemia prophylaxis during 8 cycles of BEACOPP-21 escalated or CHOP-14 chemotherapy by weekly injections of Darbepoetin 150 IU/kg. Predictions are presented in figure 11 and are compared with the actual clinical data of BEACOPP-21 escalated and CHOP-14 without EPO prophylaxis. We predict that the median minimal HB value under BEACOPP-21 escalated is 10.0 g/dl, compared to 9.0 g/dl without EPO and 10.6g/dl compared to 9.6 g/dl without EPO under CHOP-14. This would probably reduce the need for erythrocyte substitutions in these patients.

## Discussion

In the present paper, we developed a comprehensive ordinary differential equations model of human erythropoiesis under chemotherapy and EPO applications. For this purpose, we combined a former cell kinetic model of bone marrow erythropoiesis [7]–[10] with a pharmacokinetic model of EPO proposed by Krzyzanski & Wyska [13], an EPO injection model proposed by Kota *et al.* [14] and a model of chemotherapy toxicity recently developed for granulopoiesis and thrombopoiesis by our group [2], [5], [11], [12]. Since all of these models were developed in different contexts, i.e. on the basis of different data sets, it was by far not clear whether these models were compatible. The model components were kept as unchanged as possible. Adaptations necessary to combine them were discussed in detail and are often straightforward. Special emphasis is placed on the parameterisation of the model, which was performed by fitting the predictions of the model to a large variety of clinical data sets, i.e. application of different doses, schedules and derivatives of EPO with or without chemotherapy. We identified a unique parameter set which is valid for all scenarios resulting in a good explanation of the time course data of EPO serum concentration, haemoglobin, haematocrit, reticulocytes or red blood cells. We also tried to validate the model on the basis of scenarios not used for parameter fitting. We demonstrated how the model can be used to develop clinically meaningful predictions regarding supportive EPO applications during chemotherapy. Hence, the presented model of erythropoiesis completes our lineage models of haematopoiesis under chemotherapy and growth factor applications [2]–[5], [7]–[10], [12]. It was our objective to construct a model which predicts the median of patients, i.e. patients heterogeneity is not considered so far.

The proposed model is not the first attempt to model erythropoiesis: Ordinary differential equations based models of the cell kinetics of erythropoiesis in mice and rats were developed by our group in the past [6], [21], [34], [35]. Age-structured models of erythropoiesis for different purposes and applications are published in [36] (rabbit, anaemia; humans: loss of blood), [37] (humans: benzene intoxication), [38] (humans: blood donation, application of EPO Alfa), [39], [40] (mice: anaemia) and [41] (humans: blood donation). A pharmacokinetic/pharmacodynamic model of EPO applications in sheep was developed in [42], [43]. A pharmacodynamic model of EPO in rats with chemotherapy-induced anaemia was proposed in [44]. Finally, a PK/PD model of EPO applications in rats was developed in [45]. Bèlair et al. [36] provide an age-structured model of human erythropoiesis concerning recovery after blood donation regulated by endogenous EPO. The destruction rate of EPO is assumed to be constant. Exogenous EPO application or chemotherapy were not considered. The model was improved later and extended to rabbits [41]. Banks *et al.* [37] modelled the number of red blood cells under benzene intoxication in humans. The dynamics of the precursor cell population is regulated by an endogenous EPO-mediated feedback function after initial depletion of cells. Fuertinger et al. [38] developed an age-structured model of human erythropoiesis. Maturity of cells is explicitly modelled. There the system is regulated by EPO and iron status. External applications of EPO, bleeding and low oxygen partial pressure were simulated. Kota *et al.* [14] developed a model of EPO injections into sheep, which we adopted for our model. Additionally, we adopted a pharmacokinetic model of EPO applications into healthy volunteers proposed by Krzyzanski & Wyska [13]. So far, to our knowledge, none of the proposed models consider erythropoiesis under (combined) chemotherapy and EPO applications in the human situation.

The cell kinetic model of erythropoiesis proposed by Wichmann & Loeffler [9] served as a backbone of our model. Only a few adaptations were made to attach the additional model compartments to this structure. The pharmacokinetic model of EPO proposed by Krzyzanski & Wyska was attached by introducing a new influx of endogenously produced EPO. This production was modelled in accordance to Pantel [7]. EPO receptors were modelled as a function of the bone marrow content of the cell kinetic model. At this, different receptor densities of bone marrow cell stages are considered [23]. Internalised EPO of the pharmacokinetic model serves as a general input function of all feedback loops depending on EPO.

The injection model proposed by Kota *et al.* was attached in a modified version. As in [14], we assume two routes of absorption: directly and via the lymphatic system. We also assume a loss of EPO at the injection site and in the lymph compartment. But rather than assuming constant lag times, we modelled delayed absorptions by concatenated subcompartments as proposed in [11]. The efflux from the injection model serves as another influx into the central compartment of the pharmacokinetic model.

For chemotherapy modelling, we used exactly the same assumptions and equations as for our granulopoiesis model [5]. Assumptions were discussed extensively in this and in previous publications of our group (e.g. [11]). The most important assumptions are the delayed toxicity and the additivity of toxicity functions of drugs applied in combination. The first one is supported by observed bone marrow dynamics in mice after chemotherapy treatment [46]. Possible biological explanations of this phenomenon are metabolisation processes of the applied drugs, transient cell cycle arrests of cells and delayed apoptosis of cells [46]. The additivity of toxicity functions of drugs is motivated by the fact that drugs with different modes of action are combined in poly-chemotherapies. Furthermore, neglecting drug interactions is the simplest assumption from the modelling point of view requiring the lowest number of parameters. It proved to be suitable in almost all chemotherapy scenarios modelled so far including our models of granulopoiesis and thrombopoiesis. In our previous works, we encountered only a single exception not considered in this paper: The combination of Carboplatin and Paclitaxel was estimated to be clearly non-additive which is also described in clinical literature [47].

The majority of model parameters were determined by fitting the predictions of the model to clinical data sets. For this purpose, we extracted virtually all available clinical data of EPO serum concentration, haemoglobin, haematocrit, reticulocytes and red blood cells under various scenarios such as EPO application, chemotherapy and combinations of it from the literature. We also used the raw data of cooperating clinical chemotherapy trials groups. We aimed at modelling this large variety of scenarios in order to test whether our model assumptions are valid in a broader range of clinical applications.

We used the same cell kinetic parameters for all scenarios except for those quantities regulated by EPO. For these parameters, we assumed differences between Darbepoetin Alfa and other EPO derivatives. This was motivated by observed pharmacodynamic differences between Darbepoetin and conventional EPO possibly due to additional glycosylations of Darbepoetin [48]–[50]. In contrast, we assumed no differences between endogenous EPO and other EPO derivatives than Darbepoetin [51], [52]. A few cell kinetic parameters were not fitted but taken from earlier versions of our model such as normal values for amplification and transition times in BE, CE and PEB, and the parameters for the regulation of the proliferative fraction in BE. Additionally, we assumed the same parameters of stem cell regulation as in our models of granulopoiesis and thrombopoiesis in order to ensure the comparability to these models.

For parameterisation of the pharmacokinetic model, we assumed differences between Darbepoetin and conventional EPO or endogenously produced EPO again. We reparameterised the model proposed by Krzyzanski & Wyska [13] in order to achieve a better fit of other scenarios than those considered in this paper.

Since there was a large variation in injection sites for subcutaneous EPO applications, it was necessary to assume nine different parameter sets for the injection model in dependence on the site and the derivative. We aimed at keeping the number of these parameter sets as small as possible by grouping the different injection sites appropriately.

To simulate chemotherapy scenarios, it was necessary to estimate corresponding toxicity parameters. Even though the toxicity to stem cells strongly influences the model behaviour, we decided to use exactly the same estimates as for our model of granulopoiesis [5] in order to ensure comparability of the models. This also comprises the same delay parameters of chemotherapy action and the same first cycle effect. However, toxicity parameters of later cell stages still required estimation. Parameter estimates resulted in a good fit of most of the scenarios considered. No systematic deviations were observed. However, agreement was not perfect for a few scenarios. Reasons could be heterogeneity of study populations or heterogeneity of EPO assays. For chemotherapy data, we censored patients receiving erythrocyte concentrates. This was necessary to avoid biases due to high haemoglobin values after transfusion. On the other hand, this approach results in a gradual selection of patients with low haematotoxic risk. This could be a reason why haemoglobin is slightly underestimated at late therapy cycles as observed e.g. for the last cycles of the RICOVER trial (8xCHOP-14).

Parameter estimates appeared to be plausible in general. For the injection model, we estimated that EPO Beta and Darbepoetin are both characterised by a high bioavailability of about 80% (see table “Derived quantities based on parameters of the injection model” in the file S1). By the first look, this seems to be in disagreement with published results ([53]: Darbepoetin Alfa: 37%, Epoetin Alfa: 30–36%, Beta: 15–50%). However, in [53] bioavailability was defined by the ratio of the area under the curve of EPO serum concentration after subcutaneous and intravenous administration. By this definition, the bioavailability is affected by the production of EPO receptors influencing specific degradation. In contrast, we defined bioavailability as the ratio of integrated influxes into the central compartment under subcutaneous and intravenous injection respectively. These influxes are not directly observable. Applying the definition via the area under the curve, we obtain roughly the same estimates as in [53] (see table 2).

Regarding pharmacokinetic parameters, we estimated that the unspecific elimination of Darbepoetin is smaller compared to the other EPO derivatives. We also estimated that Darbepoetin is internalised more slowly. Both findings are in agreement with our biological understanding of the drug [48]–[50]. Other pharmacokinetic parameters were estimated to be similar.

We correctly predicted scenarios not used for parameter fitting. Although our validation scenarios look promising, a larger data base and a larger variety of scenarios would be required to conclusively validate our model. An optimal validation scenario would comprise closely measured time series of mature blood parameteres and EPO serum concentration after a variety of application schedules of different EPO derivatives with or without chemotherapies for which toxicity estimates are available from previous modelling.

Finally, we demonstrated how the model can be used to make clinically relevant predictions regarding the performance of EPO applications under chemotherapy. We predicted for example that the BEACOPP escalated or the CHOP-14 regimen can be supported by weekly Darbepoetin applications resulting in clear amelioration of haemoglobin decline during the course of the therapy. Our prediction can be quantitatively tested in a future clinical trial by comparing predicted and observed dynamics of haemoglobin or other parameters of red blood cell status.

We conclude that we established a model of erythropoiesis under chemotherapy and EPO applications by successfully combining four different model concepts. The model is able to explain a large number of time series data under different EPO application schedules and under chemotherapy as well. We demonstrated how the model can be used to support the planning of anaemia prophylaxis with EPO during chemotherapy. In the future, we aim at combining this model with our established models of thrombopoiesis and granulopoiesis under chemotherapy in order to construct a comprehensive model of haematologic effects of chemotherapy and growth factor applications. We also aim to include the heterogeneity of patients not considered so far.

## Supporting Information

File S1 Content: additional model equations, list of model parameters, additional model and data comparisons, sensitivity analysis
(PDF)
