# Supplementary material for: A Biomathematical Model of Human Erythropoiesis under Erythropoietin and Chemotherapy Administration
Source: PLoS One. 2013 Jun 6;8(6):e65630. doi: 10.1371/journal.pone.0065630 (PMC3675041; doi:10.1371/journal.pone.0065630)
Supplement: File S1 — Content: additional model equations, list of model parameters, additional model and data comparisons, sensitivity analysis (PDF) [file pone.0065630.s001.pdf]

A biomathematical model of human erythropoiesis under erythropoietin and chemotherapy administration

S. Schirm[1*], C. Engel[1], M. Loeffler[1], M. Scholz[1,2]

1 Institute for Medical Informatics, Statistics and Epidemiology, University of Leipzig, Leipzig, Germany

2 LIFE Research Center of Civilization Diseases, University of Leipzig, Germany

∗ E-mail: sibylle.schirm@imise.uni-leipzig.de

A Supplement Material

A.1 Important Model Variables and Mechanisms

Here, we briefly describe all major model variables and mechanisms of the cell kinetic model. We also provide all equations necessary to run the model. Since the presented model is closely related to a former model of granulopoiesis proposed by our group, details of regulation principles can be found in [1].

A.2 Cell Kinetic Model

Amplification Splitting

Influx and efflux of cells of a compartment were amplified so that the product equals the over-all amplification ($A_X^{\text{in}}(t) \cdot A_X^{\text{out}}(t) = A_X(t)$). The effect is a delayed reaction of efflux and compartment size to changes in amplification rates. It applies to all compartments with amplification, namely BE, CE, and MEB. See [1] for details.

Self renewal probability p

According to [2], this stem cell quantity is regulated by the demand of the hematopoietic bone marrow system.

$$p = F\big(C_S^{\text{rel}}(t), C_E^{\text{rel}}(t), C_G^{\text{rel}}(t), p_\delta, \theta_E, \theta_G, \theta_S\big).$$

The effect of granulopoiesis is assumed to be constant, i.e. for the present model it holds that $C_G^{\text{rel}}(t) = 1$. The parameters θ_E, θ_G, and θ_S are hypothetical weighting factors representing the influence of the com-

Table A.1. variables

quantity	meaning	type/calculation
C_X	content of compartment X function of time t	
C_X^{nor}	content of compartment X in steady state (normal value)	parameter, in general we set $C_X(0) = C_X^{nor}$
C_X^{rel}	content of compartments X relative to normal value	$C_X^{rel}(t) = \frac{C_X(t)}{C_X^{nor}}$
C_X^{in}	influx in compartment X	function of time
$C_X^{in\_nor}$	normal influx	parameter, see above
C_X^{out}	efflux from compartment X	function of time
$C_X^{out\_nor}$	normal efflux	parameter, see above
a_X	proliferative fraction in cell compartment X	function of state, sometimes constant
A_X	amplification in cell compartment X	”
A_X^{in}	amplification of influx	”
A_X^{in}	amplification of effflux	”
n_X	average number of mitoses in cell compartment X	$n_X = \text{ld } A_X$
p	self-renewal probability of stem cells	function of state
τ_X	average duration of cell cycle in compartment X	function of time, sometimes constant (not regulated)
T_X	average transit time of active cells in cell compartment X	$T_X = n_X \tau_X$
T_X^t	total transit time	$T_X^t = \frac{n_X \tau_X}{a_X}$
k	transition, degradation or toxicity coefficients	functions of time or parameter
Y^{min}	quantity Y under minimum stimulation	parameter to determine the regulatory function of Y
Y^{nor}	quantity Y in steady state	”
Y^{int}	quantity Y under intensified stimulation	”
Y^{max}	quantify Y under maximum stimulation	”
b_Y	sensitivity of Y under stimulation	”

See also [1] for details.

partments E, G, and S [1,2]. According to [2], it is assumed that

$$p_\delta = p^{nor} - p^{min} = p^{max} - p^{nor}$$

$$\theta_S(t) = \begin{cases} \frac{2}{C_S^{\text{rel}}(t)^{0.6}} & \text{for } C_S^{\text{rel}}(t) \leq 1 \\ 2 & \text{for } C_S^{\text{rel}}(t) \leq 1 \end{cases}$$

$$p = p_\delta \tanh\Big(- \theta_S(t)\big(C_S^{\text{rel}}(t) - 1\big) - \theta_E\big(C_E^{\text{rel}}(t) - 1\big) - \theta_G\big(C_G^{\text{rel}}(t) - 1\big)\Big) + 0.5,$$

assuming $C_G^{\mathrm{rel}}(t) = 1$ we have

$$p = p_\delta \tanh\Big(- \theta_S(t)\big(C_S^{\mathrm{rel}}(t) - 1\big) - \theta_E(t)\big(C_E^{\mathrm{rel}}(t) - 1\big)\Big) + 0.5\ .$$

Proliferative Fraction a_X

The proliferative fraction can be interpreted as the percentage of cells which are currently in cell cycle. These quantities are regulated by the bone marrow content.

$$a_X = F\big(C_S^{\mathrm{rel}}(t), C_E^{\mathrm{rel}}(t), C_G^{\mathrm{rel}}(t), a_X^{\min}, a_X^{\mathrm{nor}}, a_X^{\mathrm{int}}, a_X^{\max}, \omega_E, \omega_G, \omega_S\big),$$

The parameters ω are again weighting factors.

$$\begin{aligned} x = {} & \omega_E \ln C_E^{\mathrm{rel}}(t) + \omega_G \ln C_G^{\mathrm{rel}}(t) \\ & + \omega_S \begin{cases} \ln C_S^{\mathrm{rel}}(t), & \text{for} \quad C_S^{\mathrm{rel}} \leq 1 \\ C_S^{\mathrm{rel}}(t) - 1, & \text{for} \quad C_S^{\mathrm{rel}} > 1 \end{cases} \end{aligned} \tag{A.1}$$

$$\begin{aligned} y = & -\frac{1}{2\ln 2}\left(\ln\left(\frac{a_X^{\mathrm{int}} - a_X^{\max}}{a_X^{\min} - a_X^{\mathrm{int}}}\right) - \ln\left(\frac{a_X^{\mathrm{nor}} - a_X^{\max}}{a_X^{\min} - a_X^{\mathrm{nor}}}\right)\right) x \\ & + \frac{1}{2}\ln\left(\frac{a_X^{\mathrm{nor}} - a_X^{\max}}{a_X^{\min} - a_X^{\mathrm{nor}}}\right) \end{aligned}$$

$$a_X = \begin{cases} \frac{a_X^{\max} e^{-y} + a_X^{\min} e^{y}}{e^{-y} + e^{y}} & \text{for} \quad a_X^{\min} < a_X^{\mathrm{nor}} < a_X^{\mathrm{int}} < a_X^{\max} \\ a_X^{\mathrm{nor}} & \text{for} \quad a_X^{\min} = a_X^{\mathrm{nor}} = a_X^{\mathrm{int}} = a_X^{\max} \end{cases}.$$

Thus, the proliferative fraction is a monotone function ranging between $a_X^{\min}$ and $a_X^{\max}$. Low cell numbers in the bone marrow compartments cause a higher demand of proliferating cells and therefore a larger

proliferative fraction a_X. With the assumption $C_G^{\text{rel}}(t) = 1$, equation A.1 reads

$$x = \omega_E \ln C_E^{\text{rel}}(t) + \omega_S \begin{cases} \ln C_S^{\text{rel}}(t), & \text{for} \quad C_S^{\text{rel}} \leq 1 \\ C_S^{\text{rel}}(t) - 1, & \text{for} \quad C_S^{\text{rel}} > 1 \end{cases}$$

The value y defines the actual point at the regulatory curve. The variable x represents some kind of weighted logarithmic relative system size. The proliferative fraction a^{int} corresponds to $x = -\ln 2$ and a^{nor} corresponds to $x = 0$. See [1] for further details.

Stem cell compartment S

The stem cell compartment S has self-renewal capability. Under steady state conditions, 50% of the cells which arise from S remain in this compartment, the others feed the BE compartment.

$$\frac{d}{dt} C_S = (2p - 1) C_S \frac{a_S}{\tau_S} - \Psi_S \cdot C_S \tag{A.2}$$

$$C_S^{\text{out}} = 2(1 - p) C_S \frac{a_S}{\tau_S} \tag{A.3}$$

where Ψ_S is the summarized chemotherapy function. It holds that $p^{\text{nor}} = \frac{1}{2}$. Thus, for the initial conditions it holds that

$$C_S(0) = C_S^{\text{nor}} = 1 \tag{A.4}$$

$$C_S^{\text{out}}(0) = C_S^{\text{out\_nor}} = 2(1 - p^{\text{nor}}) C_S^{\text{nor}} \frac{a_S^{\text{nor}}}{\tau_S}. \tag{A.5}$$

Compartment BE

$$\frac{d}{dt} C_{\text{BE}} = \alpha_E C_S^{\text{out}} A_{\text{BE}}^{\text{in}} - C_{\text{BE}} \frac{a_{\text{BE}}}{\tau_{\text{BE}}} - \Psi_{\text{BE}} \cdot C_{\text{BE}}$$

$$C_{\text{BE}}^{\text{out}} = C_{\text{BE}} A_{\text{BE}}^{\text{out}} \frac{a_{\text{BE}}}{T_{\text{BE}}}$$

with the initial conditions

$$C_{\mathrm{BE}}(0) = C_{\mathrm{BE}}^{\mathrm{nor}} = \alpha_E C_S^{\mathrm{out\_nor}} A_{\mathrm{BE}}^{\mathrm{in\_nor}} \frac{T_{\mathrm{BE}}^{\mathrm{nor}}}{a_{\mathrm{BE}}^{\mathrm{nor}}}$$
$$C_{\mathrm{BE}}^{\mathrm{out\_nor}} = C_{\mathrm{BE}}^{\mathrm{nor}} A_{\mathrm{BE}}^{\mathrm{out\_nor}} \frac{a_{\mathrm{BE}}^{\mathrm{nor}}}{T_{\mathrm{BE}}^{\mathrm{nor}}}$$
$$= \alpha_E C_S^{\mathrm{out\_nor}} A_{\mathrm{BE}}^{\mathrm{nor}}.$$

Compartment CE

$$A_{\mathrm{CE}} = Z(C_{EPO}^{\mathrm{rel}})$$
$$\frac{d}{dt} C_{\mathrm{CE}} = C_{\mathrm{BE}}^{\mathrm{out}} A_{\mathrm{CE}}^{\mathrm{in}} - C_{\mathrm{CE}} \frac{a_{\mathrm{CE}}}{T_{\mathrm{CE}}} - \Psi_{\mathrm{CE}} \cdot C_{\mathrm{CE}}$$
$$C_{\mathrm{CE}}^{\mathrm{out}} = C_{\mathrm{CE}} A_{\mathrm{CE}}^{\mathrm{out}} \frac{a_{\mathrm{CE}}}{T_{\mathrm{CE}}}.$$

We assume $a_{\mathrm{CE}} = 1$. Thus,

$$C_{\mathrm{CE}}(0) = C_{\mathrm{CE}}^{\mathrm{nor}} = C_{\mathrm{BE}}^{\mathrm{out\_nor}} A_{\mathrm{CE}}^{\mathrm{in\_nor}} T_{\mathrm{CE}}^{\mathrm{nor}}$$
$$C_{\mathrm{CE}}^{\mathrm{out}}(0) = C_{\mathrm{CE}}^{\mathrm{out\_nor}} = C_{\mathrm{BE}}^{\mathrm{out\_nor}} A_{\mathrm{CE}}^{\mathrm{nor}}.$$

Compartment PEB

$$A_{\mathrm{PEB}} = Z(C_{EPO}^{\mathrm{rel}})$$
$$\frac{d}{dt} C_{\mathrm{PEB}} = C_{\mathrm{CE}}^{\mathrm{out}} A_{\mathrm{PEB}}^{\mathrm{in}} - C_{\mathrm{PEB}} \frac{a_{\mathrm{PEB}}}{T_{\mathrm{PEB}}} - \Psi_{\mathrm{PEB}} \cdot C_{\mathrm{PEB}}$$
$$C_{\mathrm{PEB}}^{\mathrm{out}} = C_{\mathrm{PEB}} A_{\mathrm{PEB}}^{\mathrm{out}} \frac{a_{\mathrm{PEB}}}{T_{\mathrm{PEB}}}.$$

We assume $a_{\mathrm{PEB}} = 1$. Thus,

$$C_{\mathrm{PEB}}(0) = C_{\mathrm{PEB}}^{\mathrm{nor}} = C_{\mathrm{CE}}^{\mathrm{out\_nor}} A_{\mathrm{PEB}}^{\mathrm{in\_nor}} \frac{T_{\mathrm{PEB}}^{\mathrm{nor}}}{a_{\mathrm{PEB}}^{\mathrm{nor}}}$$
$$C_{\mathrm{PEB}}^{\mathrm{out}}(0) = C_{\mathrm{PEB}}^{\mathrm{out\_nor}} = C_{\mathrm{CE}}^{\mathrm{out\_nor}} A_{\mathrm{PEB}}^{\mathrm{nor}}.$$

Compartment MEB

The maturation is modeled by splitting MEB into $N_{\mathrm{MEB}} = 15$ subcompartments without amplification.

$$\begin{aligned}
T_{\mathrm{MEB}} &= Z(C^{\mathrm{rel}}_{EPO}) \\
C_{\mathrm{MEB}} &= \sum_{i=1}^{N_{\mathrm{MEB}}} C_{\mathrm{MEB}_i} \\
\frac{d}{dt} C_{\mathrm{MEB}_1} &= C^{\mathrm{out}}_{\mathrm{PEB}} - C_{\mathrm{MEB}_1} \frac{N_{\mathrm{MEB}}}{T_{\mathrm{MEB}}} - \Psi_{\mathrm{MEB}} \cdot C_{\mathrm{MEB}_1} \\
\frac{d}{dt} C_{\mathrm{MEB}_i} &= C^{\mathrm{out}}_{\mathrm{MEB}_{i-1}} - C_{\mathrm{MEB}_i} \frac{N_{\mathrm{MEB}}}{T_{\mathrm{MEB}}} - \Psi_{\mathrm{MEB}} \cdot C_{\mathrm{MEB}_i}, \quad i = 2, \ldots, N_{\mathrm{MEB}} \\
C^{\mathrm{out}}_{\mathrm{MEB}_i} &= C_{\mathrm{MEB}_i} \frac{N_{\mathrm{MEB}}}{T_{\mathrm{MEB}}}, \quad i = 1, \ldots, N_{\mathrm{MEB}} \\
C^{\mathrm{out}}_{\mathrm{MEB}} &= C^{\mathrm{out}}_{\mathrm{MEB}_{N_{\mathrm{MEB}}}},
\end{aligned}$$

with the initial values

$$\begin{aligned}
C_{\mathrm{MEB}}(0) &= C^{\mathrm{nor}}_{\mathrm{MEB}} = C^{\mathrm{out_nor}}_{\mathrm{PEB}} T^{\mathrm{nor}}_{\mathrm{MEB}} \\
C_{\mathrm{MEB}_i}(0) &= C^{\mathrm{nor}}_{\mathrm{MEB}_i} = C^{\mathrm{out_nor}}_{\mathrm{PEB}} \frac{T^{\mathrm{nor}}_{\mathrm{MEB}}}{N_{\mathrm{MEB}}}, \quad i = 1, \ldots, N_{\mathrm{MEB}} \\
C^{\mathrm{out}}_{\mathrm{MEB}_i}(0) &= C^{\mathrm{out_nor}}_{\mathrm{MEB}_i} = C^{\mathrm{nor}}_{\mathrm{MEB}_i} \frac{N_{\mathrm{MEB}}}{T^{\mathrm{nor}}_{\mathrm{MEB}}} = C^{\mathrm{out_nor}}_{\mathrm{PEB}}, \quad i = 1, \ldots, N_{\mathrm{MEB}} \\
C^{\mathrm{out}}_{\mathrm{MEB}}(0) &= C^{\mathrm{out_nor}}_{\mathrm{MEB}} = C^{\mathrm{out_nor}}_{\mathrm{MEB}_{N_{\mathrm{MEB}}}} = C^{\mathrm{out_nor}}_{\mathrm{PEB}}.
\end{aligned}$$

Compartment RET

$$\begin{aligned}
T_{\mathrm{RET}} &= T^{\mathrm{nor}}_{\mathrm{MEB}} + T^{\mathrm{nor}}_{\mathrm{RET}} - T_{\mathrm{MEB}} \\
\frac{d}{dt} C_{\mathrm{RET}} &= C^{\mathrm{out}}_{\mathrm{MEB}} - \frac{C_{\mathrm{RET}}}{T_{\mathrm{RET}}} \\
C^{\mathrm{out}}_{\mathrm{RET}} &= \frac{C_{\mathrm{RET}}}{T_{\mathrm{RET}}} \\
C_{\mathrm{RET}}(0) &= C^{\mathrm{nor}}_{\mathrm{RET}} = C^{\mathrm{nor}}_{\mathrm{ERY}} \frac{q_{\mathrm{RET}}}{1 - q_{\mathrm{RET}}} \\
C^{\mathrm{out}}_{\mathrm{RET}}(0) &= C^{\mathrm{out_nor}}_{\mathrm{RET}} = C^{\mathrm{out_nor}}_{\mathrm{MEB}} \\
T^{\mathrm{nor}}_{\mathrm{RET}} &= \frac{C^{\mathrm{nor}}_{\mathrm{RET}}}{C^{\mathrm{out_nor}}_{\mathrm{RET}}} = \frac{q_{\mathrm{RET}}}{1 - q_{\mathrm{RET}}} \big((1 - s^{\mathrm{nor}}_{\mathrm{ERY}}) T_{\mathrm{ERY_rnd}} + s^{\mathrm{nor}}_{\mathrm{ERY}} T_{\mathrm{ERY_age}} \big).
\end{aligned}$$

q_{RET} is the ratio of reticulocytes to the total number of red blood cells in steady state. $s_{\mathrm{ERY}}^{\mathrm{nor}}$, $T_{\mathrm{ERY\_rnd}}$, and $T_{\mathrm{ERY\_age}}$ are explained in the next section.

Compartment ERY

The compartment ERY is split into the subcompartments "RANDOM" and "AGE". In steady state, most erythrocytes die dependent on age. The age dependent reduction is modeled by division into subcompartments. Under stimulation, the apoptosis is more randomly (see [3]). To model this observation, the influxes into the subcompartments "RANDOM" and "AGE" are regulated by the factor s_{ERY}, which depends on the bone marrow output of the reticulocytes. $T_{\mathrm{ERY\_rnd}}$, and $T_{\mathrm{ERY\_age}}$ are the corresponding transition times of these compartments (see [1–4] for details.)

$$
\begin{aligned}
s_{\mathrm{ERY}} &= \exp\left(\left(\frac{C_{\mathrm{RET}}^{\mathrm{out}}}{C_{\mathrm{RET}}^{\mathrm{out\_nor}}}\right)^2 \ln s_{\mathrm{ERY}}^{\mathrm{nor}}\right)\\
C_{\mathrm{ERY}} &= C_{\mathrm{ERY\_age}} + C_{\mathrm{ERY\_rnd}}\\
C_{\mathrm{ERY\_age}} &= \sum_{i=1}^{N_{\mathrm{ERY}}} C_{\mathrm{ERY\_age\_}i}\\
\frac{d}{dt}C_{\mathrm{ERY\_age\_1}} &= s_{\mathrm{ERY}} C_{\mathrm{RET}}^{\mathrm{out}} - C_{\mathrm{ERY\_age\_1}}\frac{N_{\mathrm{ERY}}}{T_{\mathrm{ERY\_age}}}\\
\frac{d}{dt}C_{\mathrm{ERY\_age\_}i} &= C_{\mathrm{ERY\_age\_}(i-1)}^{\mathrm{out}} - C_{\mathrm{ERY\_age\_}i}^{\mathrm{out}}, \quad i = 2,\ldots,N_{\mathrm{ERY}}\\
C_{\mathrm{ERY\_age\_}i}^{\mathrm{out}} &= C_{\mathrm{ERY\_age\_}i}\frac{N_{\mathrm{ERY}}}{T_{\mathrm{ERY\_age}}}\\
\frac{d}{dt}C_{\mathrm{ERY\_rnd}} &= (1 - s_{\mathrm{ERY}})C_{\mathrm{RET}}^{\mathrm{out}} - C_{\mathrm{ERY\_rnd}}\frac{1}{T_{\mathrm{ERY\_rnd}}},
\end{aligned}
$$

with initial conditions

$$
\begin{aligned}
C_{\mathrm{ERY}}(0) &= C_{\mathrm{ERY}}^{\mathrm{nor}} = C_{\mathrm{ERY\_age}}^{\mathrm{nor}} + C_{\mathrm{ERY\_rnd}}^{\mathrm{nor}}\\
C_{\mathrm{ERY\_age}}(0) &= C_{\mathrm{ERY\_age}}^{\mathrm{nor}} = \sum_{i=1}^{N_{\mathrm{ERY}}} C_{\mathrm{ERY\_age\_}i}^{\mathrm{nor}} = s_{\mathrm{ERY}}^{\mathrm{nor}} C_{\mathrm{RET}}^{\mathrm{out\_nor}} T_{\mathrm{ERY\_age}}\\
C_{\mathrm{ERY\_age\_1}}(0) &= C_{\mathrm{ERY\_age\_1}}^{\mathrm{nor}} = s_{\mathrm{ERY}}^{\mathrm{nor}} C_{\mathrm{RET}}^{\mathrm{out\_nor}}\frac{T_{\mathrm{ERY\_age}}}{N_{\mathrm{ERY}}}
\end{aligned}
$$

$$C_{\text{ERY\_age\_}i}(0) = C^{\text{nor}}_{\text{ERY\_age\_}i} = C^{\text{out\_nor}}_{\text{ERY\_age\_}i-1}\frac{T_{\text{ERY\_age}}}{N_{\text{ERY}}}, \quad i = 2, \dots, N_{\text{ERY}}$$

$$= s^{\text{nor}}_{\text{ERY}} C^{\text{out\_nor}}_{\text{RET}} \frac{T_{\text{ERY\_age}}}{N_{\text{ERY}}}, \quad i = 1, \dots, N_{\text{ERY}}$$

$$C^{\text{out}}_{\text{ERY\_age\_}i}(0) = C^{\text{out\_nor}}_{\text{ERY\_age\_}i} = C^{\text{nor}}_{\text{ERY\_age\_}i}\frac{N_{\text{ERY}}}{T_{\text{ERY\_age}}} = s^{\text{nor}}_{\text{ERY}} C^{\text{out\_nor}}_{\text{RET}}$$

$$C_{\text{ERY}_{\text{rnd}}}(0) = C^{\text{nor}}_{\text{ERY\_rnd}} = (1 - s^{\text{nor}}_{\text{ERY}}) C^{\text{out\_nor}}_{\text{RET}} T_{\text{ERY}_{\text{rnd}}}$$

Endogenous production of EPO

The endogenous production of EPO (EPO_{prod}) is assumed to be dependent on the tissue oxygen tension in the kidneys and the number of circulating red blood cells (see [4, 5]). Pantel [4] and Wichmann [5] proposed the following model of this process.

Table A.2. Variables for endogenous EPO production

quantity	meaning	type/calculation	
$P^t_{O_2}$	tissue oxygen tension in kidneys	function of time	[4, 5]
$P^{t\_\text{nor}}_{O_2}$	normal value of tissue oxygen tension in kidneys		[4, 5]
$S^t_{O_2}$	tissue saturation of oxygen	function of time	[4, 5]
$S^{t\_\text{nor}}_{O_2}$	normal tissue saturation of oxygen	constant	[4, 5]
P_{50}	partial oxygen pressure corresponding to $S^t_{O_2} = 50\%$	26.5 mm Hg	[4, 5]
$P^{A\_\text{nor}}_{O_2}$	arterial oxygen tension, normal value	97 mm Hg	[4, 5]
ΔSO_2	desaturation of HB (arteriovenous difference), normal value	20 %	[4, 5]
γ	Hill coefficient	2.65	[4, 5]
P^{endo}_{max}	maximum EPO production	200 (set)	[4, 5]
b_{EPO}	sensitivity of EPO production to changes in $P^t_{O_2}$	ln 200 (set)	[4, 5]

$$P^t_{O_2} = P_{50} \cdot \left(\frac{S^t_{O_2}}{100 - S^t_{O_2}}\right)^{\frac{1}{\gamma}} \text{(Hill equation)} \tag{A.6}$$

$$S^t_{O_2} = \frac{100}{\left(\frac{P_{50}}{P^{A\_\text{nor}}_{O_2}}\right)^{\gamma} + 1} - \Delta SO_2 \cdot \frac{RET^{\text{nor}} + ERY^{\text{nor}}}{C_{\text{RET}} + C_{\text{ERY}}} \tag{A.7}$$

$$S^{t\_\text{nor}}_{O_2} = \frac{100}{\left(\frac{P_{50}}{P^{A\_\text{nor}}_{O_2}}\right)^{\gamma} + 1} - \Delta SO_2 \tag{A.8}$$

$$P^{t\_\text{nor}}_{O_2} = P_{50} \cdot \left(\frac{S^{t\_\text{nor}}_{O_2}}{100 - S^{t\_\text{nor}}_{O_2}}\right)^{\frac{1}{\gamma}} \tag{A.9}$$

Define $f = \frac{P^t_{O_2}}{P^{t\_nor}_{O_2}}$, we assume

$$\begin{aligned} \mathrm{EPO_{prod}} &= P^{\mathrm{endo}}_{max} \cdot e^{-b_{\mathrm{EPO}} \cdot f} \qquad [4] \\ \mathrm{EPO_{prod}}(0) &= 1. \end{aligned} \tag{A.10}$$

For further explanations and justifications, see [4,5].

A.3 List of Model Parameters

parameter	value			
q_{RET}	0.016		set	
$T^{\mathrm{rnd}}_{\mathrm{ERY}}$	1020.4	h	set	[6]
$T^{\mathrm{age}}_{\mathrm{ERY}}$	3061.2	h	set	[6]
$s^{\mathrm{nor}}_{\mathrm{ERY}}$	0.900		set	[4], p. 40
N_{ERY}	10.0		set	[4], p. 41
$\mathrm{HCT}^{\mathrm{nor}}$	0.430		set	
$\mathrm{ERY}^{\mathrm{nor}}$	4.50		set	
$\mathrm{RET}^{\mathrm{nor}}$	100	x1000/μl	set	
$\mathrm{RET\%}^{\mathrm{nor}}$	9.50		set	
$\mathrm{HB}^{\mathrm{nor}}$	13.5		set	
P^{endo}_{max}	200		set	[4]
b_{EPO}	5.30		fitted	
$\mathrm{EPO_{Vc}}$	0.0320	l/kg	set	[7]
$\mathrm{EPO_{serum}}$	15	IU/l	set	[7]
$a^{\mathrm{min}}_{\mathrm{BE}}$	0.30		set	[2], p. 71
$a^{\mathrm{nor}}_{\mathrm{BE}}$	0.33		set	[2], p. 71
$a^{\mathrm{int}}_{\mathrm{BE}}$	0.66		set	[2], p. 71
$a^{\mathrm{max}}_{\mathrm{BE}}$	1.00		set	[2], p. 71
α_G	0.8		set	[2], p. 73
α_E	0.15		set	[2], p. 73
S^{nor}	1		set	
τ_S	8		set	[2], p. 70
a^{min}_S	0.01		set	[2], p. 70
p_δ	0.1		set	[2], p. 70
a^{nor}_S	0.15		set	[2], p. 70
a^{int}_S	0.45		set	[2], p. 70
a^{max}_S	1		set	[2], p. 70
w_E	0.3		set	[2], p. 70
w_G	0.1		set	[2], p. 70
w_S	1		set	[2], p. 70
ϑ_E	-2		set	[2], p. 70
ϑ_G	-8		set	[2], p. 70

Table A.3. EPO PK/PD parameters

	Alfa, Beta, Delta, endogenous EPO				Darbepoetin Alfa	
k_{el}	0.102		fitted		0.062	fitted
k_{12}	0.079		fitted		0.294	fitted
k_{21}	0.084		fitted		0.291	fitted
k_{on}	0.070		set	[7]	0.043	fitted
k_{off}	14.27		set	[7]	9.62	fitted
R_0	64.31		set	[7]	64.31	set
k_{int}	2		set	[7]	1.14	fitted
k_{deg}	0.101		set	[7]	0.116	fitted
w_{RET}	0.05		set		0.05	set
w_{MEB}	0.087		fitted		0.125	fitted
w_{PEB}	0.293		fitted		0.509	fitted
w_{CE}	3.84		fitted		2.69	fitted
w_{BE}	0.0881		fitted		0.111	fitted
$k_{\mathrm{on}}/k_{\mathrm{off}}$	0.004875				0.004453	
T_{BE}^{min}	155.7	h	fitted		124.6	fitted
T_{BE}^{nor}	40	h	set	[6]	40	set
T_{BE}^{max}	28.60	h	fitted		34.22	fitted
T_{BE}^{b}	1.134		fitted		3.559	fitted
A_{BE}^{min}	25.04		fitted		47.10	fitted
A_{BE}^{nor}	64		set	[6]	64	set
A_{BE}^{max}	194.7		fitted		115.5	fitted
A_{BE}^{b}	2.321		fitted		0.1659	fitted
A_{CE}^{min}	0.9645		fitted		0.5717	fitted
A_{CE}^{nor}	32		set	[6]	32	set
A_{CE}^{max}	104.7		fitted		127.8	fitted
A_{CE}^{b}	0.0438		fitted		0.3956	fitted
T_{CE}^{min}	186.7	h	fitted		387.3	fitted
T_{CE}^{nor}	40	h	set	[6]	40	set
T_{CE}^{max}	15.25	h	fitted		36.23	fitted
T_{CE}^{b}	0.3920		fitted		0.1305	fitted
T_{PEB}^{min}	99.25	h	fitted		178.4	fitted
T_{PEB}^{nor}	48	h	set		48	set
T_{PEB}^{max}	8.809	h	fitted		15.74	fitted
T_{PEB}^{b}	1.301		fitted		1.847	fitted
A_{PEB}^{min}	0.6862		fitted		0.8078	fitted
A_{PEB}^{nor}	64		set	[6]	64	set
A_{PEB}^{max}	75.38		fitted		139.6	fitted
A_{PEB}^{b}	0.4135		fitted		0.1331	fitted
T_{MEB}^{min}	144.8	h	fitted		186.7	fitted
T_{MEB}^{nor}	100.2	h	fitted		100.2	fitted
T_{MEB}^{max}	90.17	h	fitted		26.98	fitted
T_{MEB}^{b}	0.5395		fitted		0.1965	fitted

Table A.4. EPO absorption parameters after subcutaneous injection (fitted)

	Alfa, abdomen, upper arm	Alfa, fore-arm	Alfa, shoulder	Alfa, thigh	Beta, abdomen	Beta, thigh	Beta, fore-arm	Delta	Darb-epoetin Alfa
k_a^F	0.5320	0.7786	0.7119	0.7374	0.3747	0.4528	0.4077	0.6657	3.0148
k_e^F	0.2713	0.1337	0.1263	0.3295	0.1062	0.0656	0.1873	0.1517	0.1938
k_{Delay}^L	0.0390	0.0510	0.1460	0.0298	0.0699	0.0410	0.0476	0.2367	0.0241
k_a^L	0.1172	0.0901	0.1326	0.1588	0.2309	0.2024	0.4687	0.1065	1.1192
k_e^L	0.4334	0.4904	0.4066	0.4343	0.0950	0.0557	0.1587	0.3321	0.1769
k_{Delay}^F	0.3275	0.3259	0.6029	0.1626	0.3705	0.2078	0.2819	0.7508	0.1161
k_{FL}	1.0074	3.6252	3.9596	0.7213	1.2138	1.0453	1.1987	3.4461	5.5404

Table A.5. Toxicity parameters of Chemotherapies

drug or drug combination	Therapy	FC	Delay	S	BE	CE	PEB	MEB	RET
Cyclophosphamid 650 mg/m^2 Doxorubicin 25 mg/m^2	BEACOPP	1.111	0.064	0.001	0.000	0.000	0.005	0.000	0.002
Cyclophosphamid 750 mg/m^2 Doxorubicin 50 mg/m^2	CHOP	1.111	0.064	0.216	0.000	0.017	0.006	0.117	0.036
Cyclophosphamid 1250 mg/m^2 Doxorubicin 35 mg/m^2	BEACOPP escalated	1.111	0.064	0.212	0.001	0.001	0.016	0.030	0.021
Cyclophosphamid 1400 mg/m^2 Doxorubicin 32.5 mg/m^2	high CHOEP	1.111	0.064	0.194	0.002	0.021	0.063	0.117	0.067
Etoposid 100 mg/m^2	CHOEP	1.097	0.068	0.000	0.000	0.000	0.041	0.000	0.011
Etoposid 200 mg/m^2	BEACOPP escalated	1.097	0.068	0.000	0.000	0.000	0.057	0.013	0.020
Etoposid 175 mg/m^2	high CHOEP	1.097	0.068	0.024	0.001	0.000	0.044	0.000	0.026
Procarbazine 100 mg/m^2	BEACOPP escalated, BEACOPP	1.092	0.013	0.002	0.011	0.004	0.013	0.000	0.000
Bleomycin 10 mg/m^2	BEACOPP escalated, BEACOPP	1.323	0.003	0.012	0.010	0.011	0.001	0.030	0.000
Platinum Etoposide	Platinum + Etoposide	1.000	1.219	0.338	0.011	0.002	0.008	0.007	0.000
Paclitaxel 225 mg/m^2	ETC	1.050	0.017	0.246	0.000	0.436	1.748	0.000	0.096
Cyclophosphamid 2500 mg/m^2	ETC	1.005	0.064	0.199	0.033	0.056	0.059	0.000	0.267
Epirubicin 150 mg/m^2	ETC	1.988	0.045	0.003	0.005	1.506	2.909	0.022	3.273
Cyclophosphamid 600 mg/m^2	ECT	1.005	0.064	0.199	0.003	0.008	0.016	0.000	0.045
Epirubicin 90 mg/m^2	ECT	1.988	0.045	0.000	0.000	0.041	0.179	0.001	0.174
Paclitaxel 175 mg/m^2	ECT	1.050	0.017	0.000	0.000	0.167	0.113	0.000	0.040

Table A.6. Derived quantities based on parameters of the injection model

		bioavailability
Alfa	abdomen, upper arm	0.4123
Alfa	shoulder	0.3513
Alfa	forearm	0.2957
Alfa	thigh	0.5204
Beta	abdomen	0.7286
Beta	thigh	0.8137
Beta	forearm	0.7266
Delta		0.3524
Darbepoetin		0.8914

A.4 Sensitivity Analysis of Model Parameters

We analyzed the sensitivity of the model parameters in the following way. The parameters were increased or decreased by 2.5 % and the change of the sum of the fitness functions as percentage is plotted as bar diagrams. Values of the fitness functions of different scenarios are added.

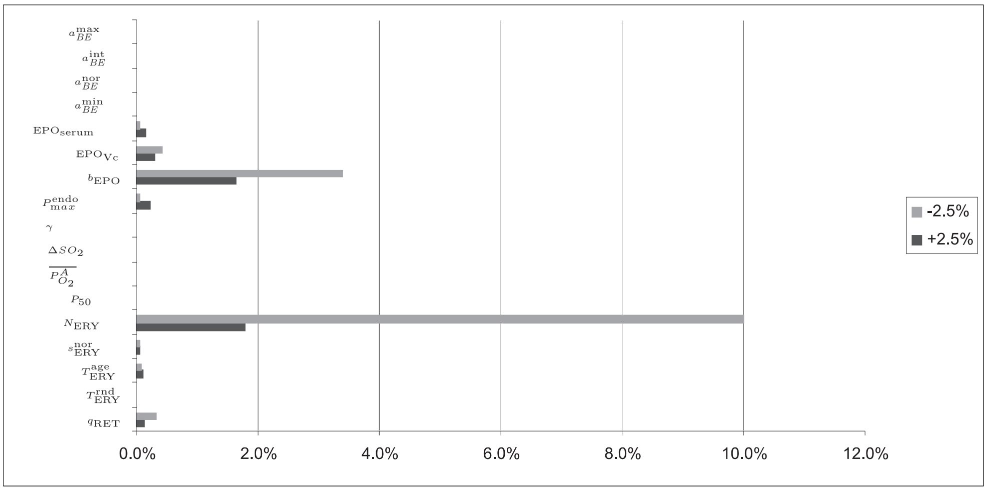

Figure 1. Sensitivity of parameters used for EPO Alfa, Beta, Delta, and Darbepoetin Alfa

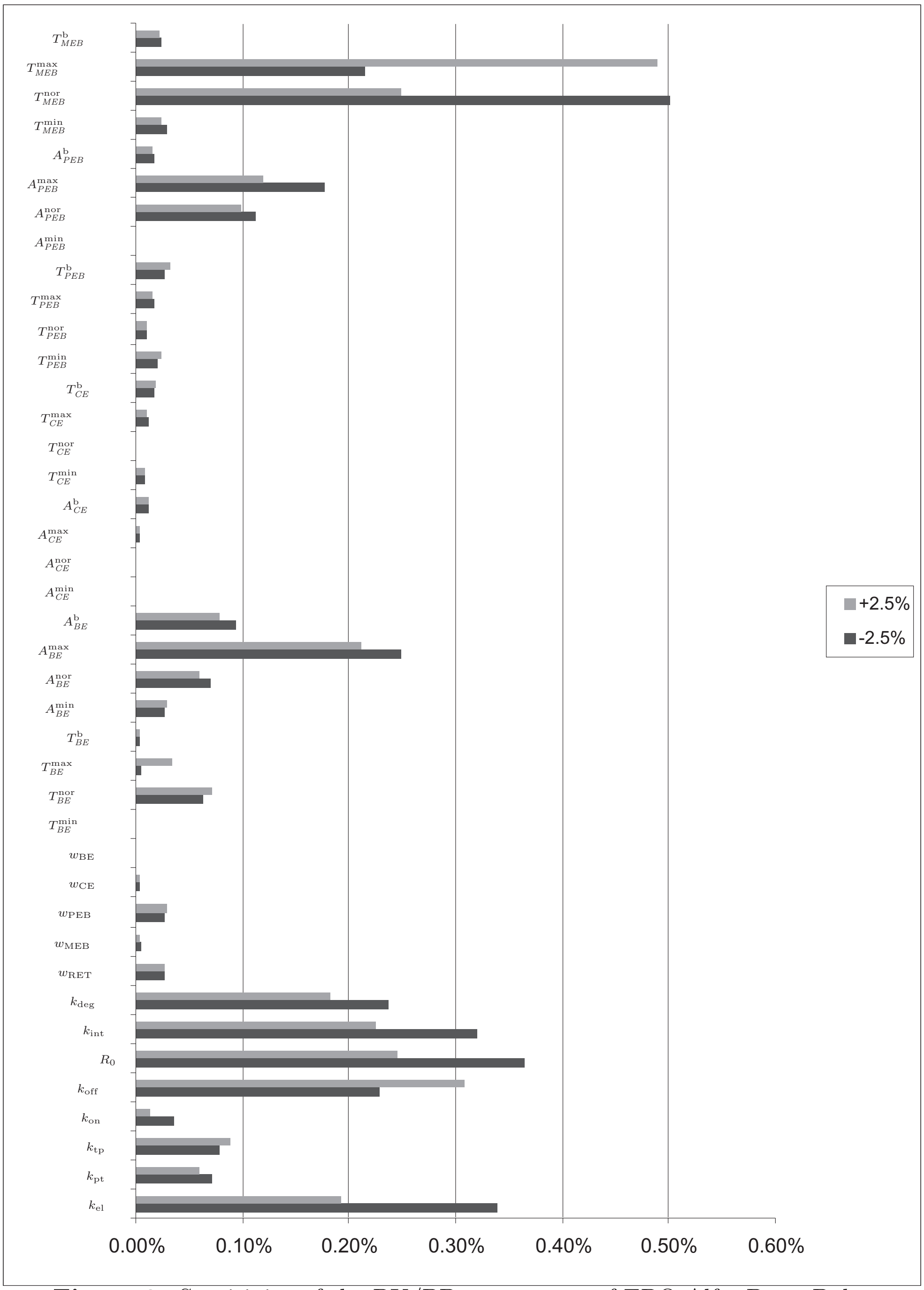

Figure 2. Sensitivity of the PK/PD parameters of EPO Alfa, Beta, Delta

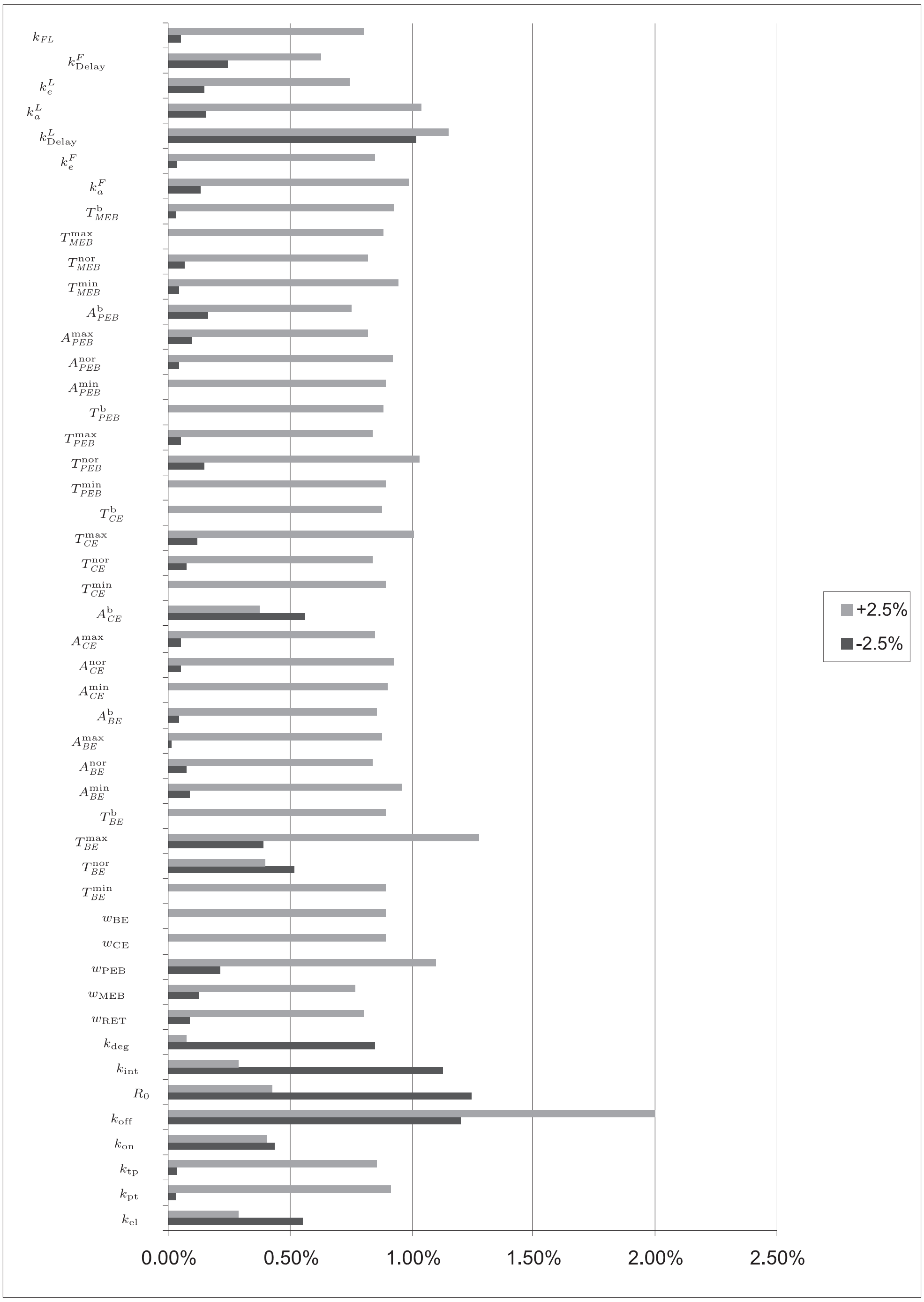

Figure 3. Sensitivity of the PK/PD parameters for Darbepoetin.

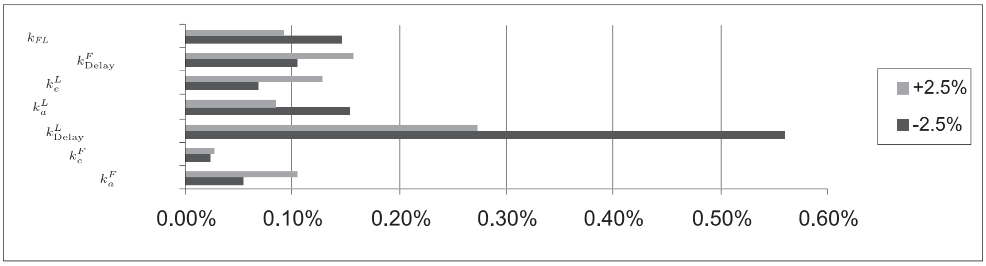

Figure 4. Sensitivity of the different parameters for subcutaneous injection of EPO Alfa, Beta, Delta

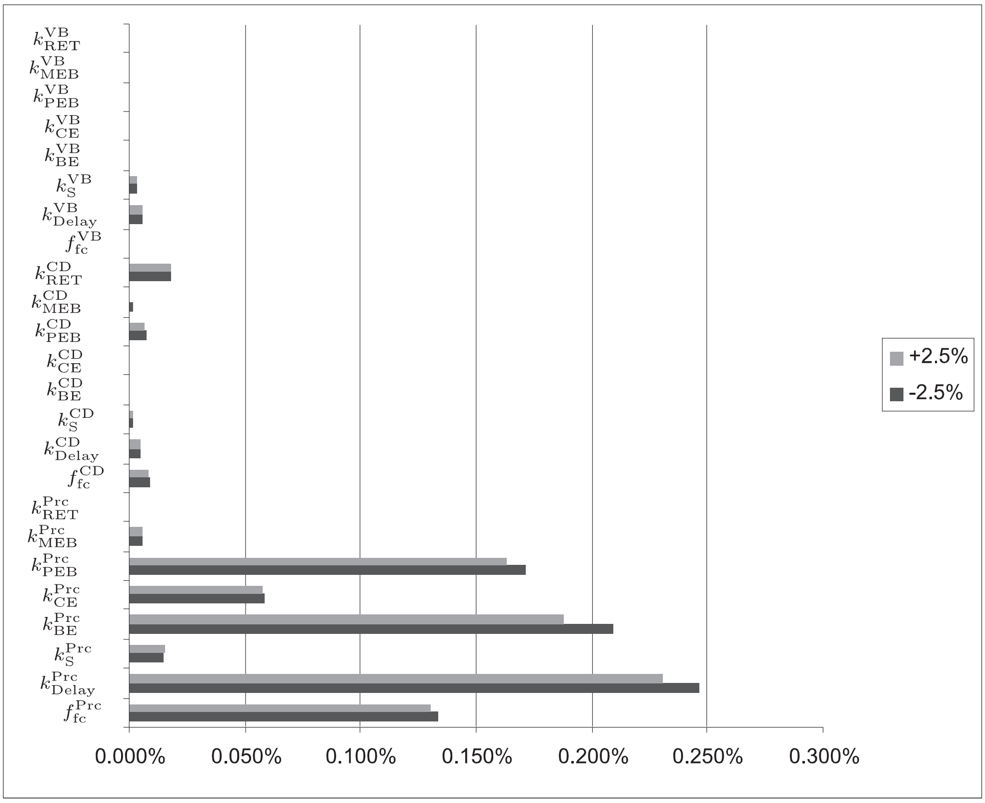

Figure 5. Sensitivity of toxicity parameters for BEACOPP chemotherapy

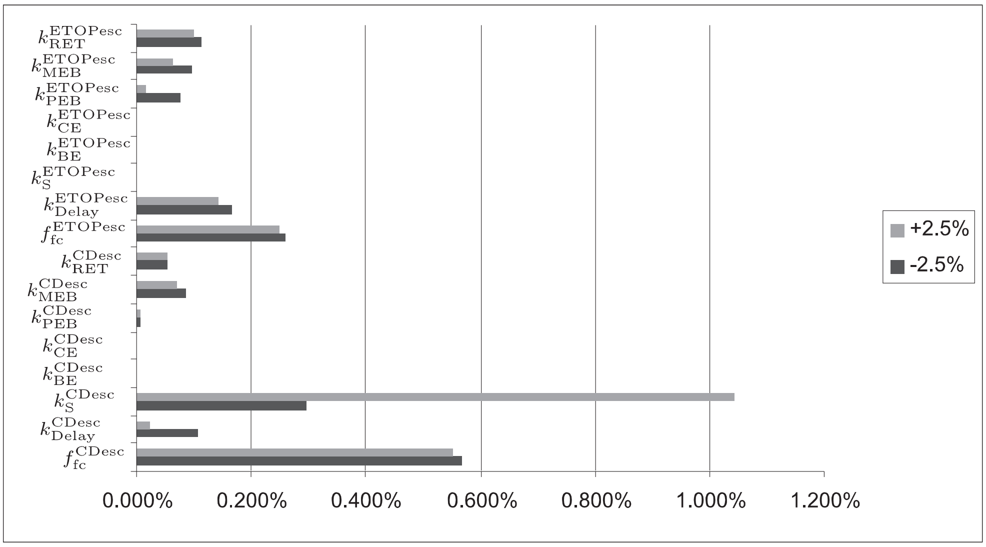

Figure 6. Sensitivity of toxicity parameters for BEACOPP escalated chemotherapy

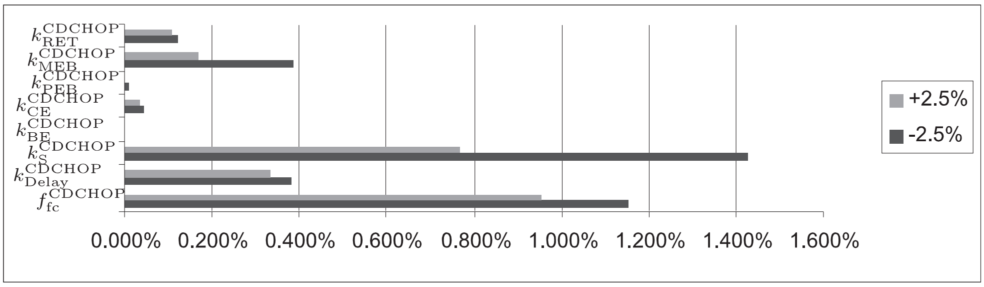

Figure 7. Sensitivity of toxicity parameters for CHOP chemotherapy

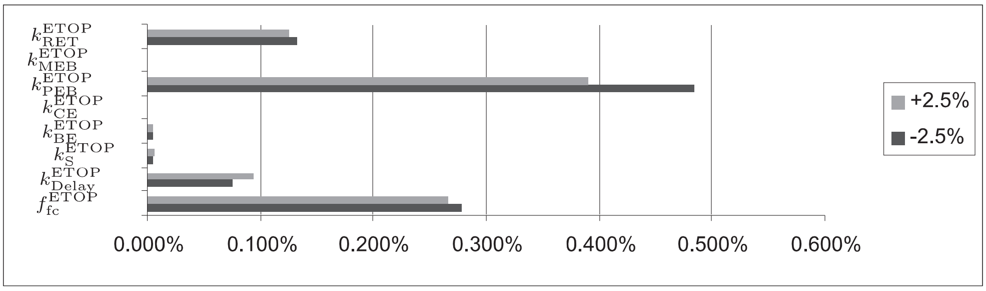

Figure 8. Sensitivity of toxicity parameters for etoposide

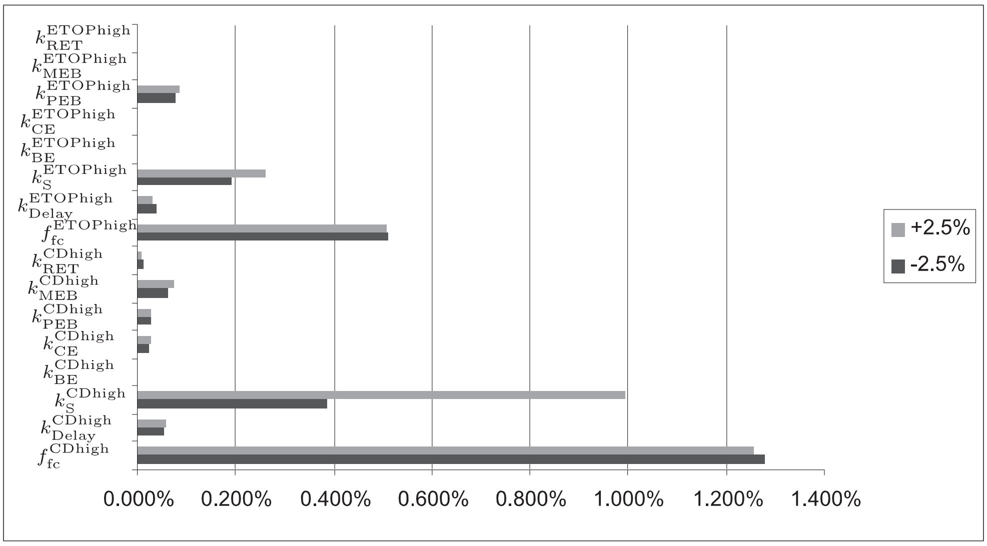

Figure 9. Sensitivity of toxicity parameters for high-CHOEP chemotherapy

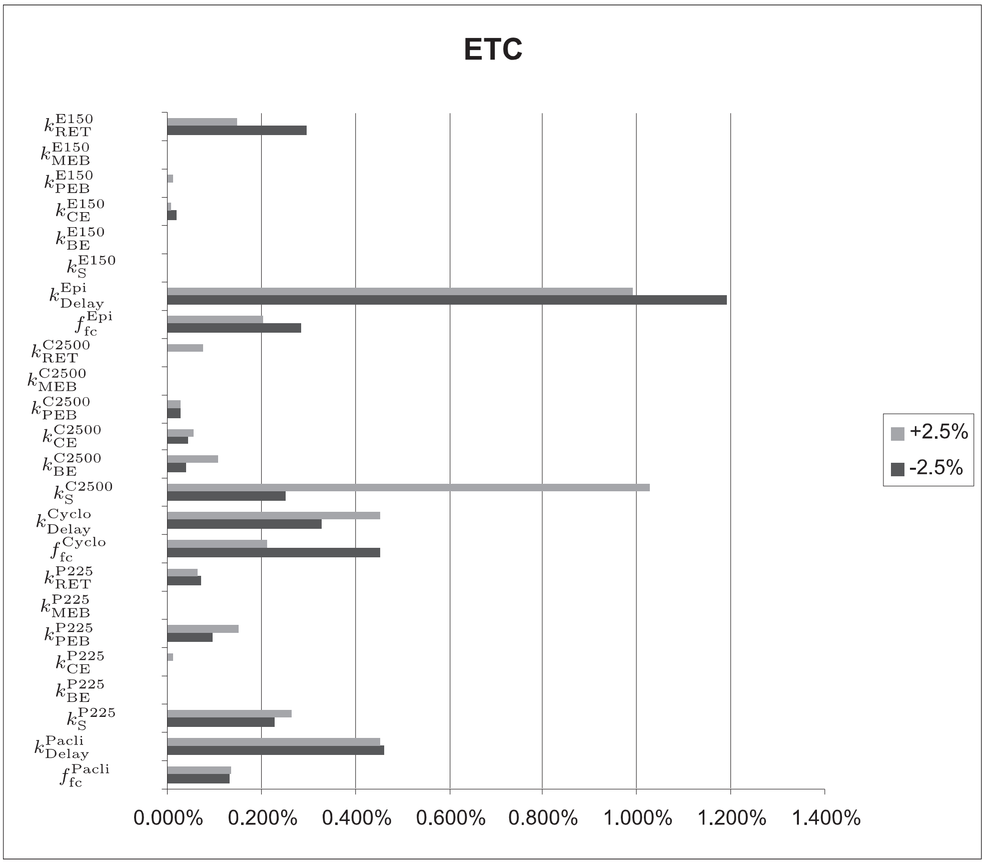

Figure 10. Sensitivity of toxicity parameters for ETC chemotherapy

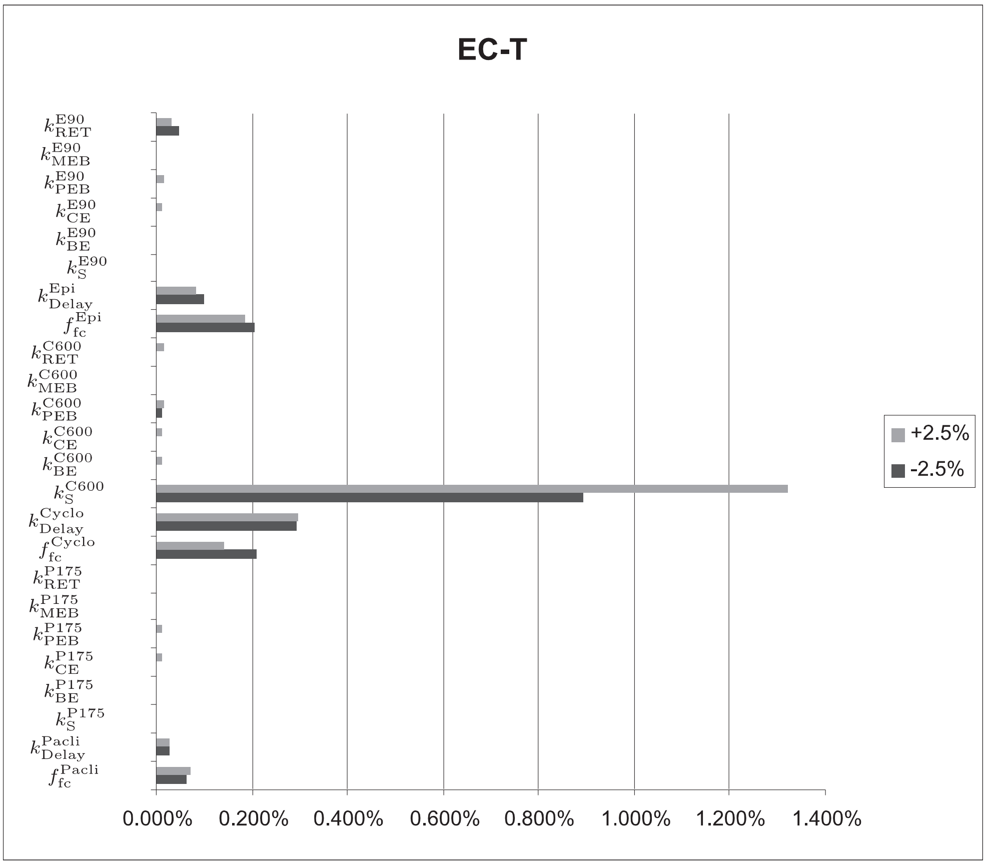

Figure 11. Sensitivity of toxicity parameters for EC-T chemotherapy

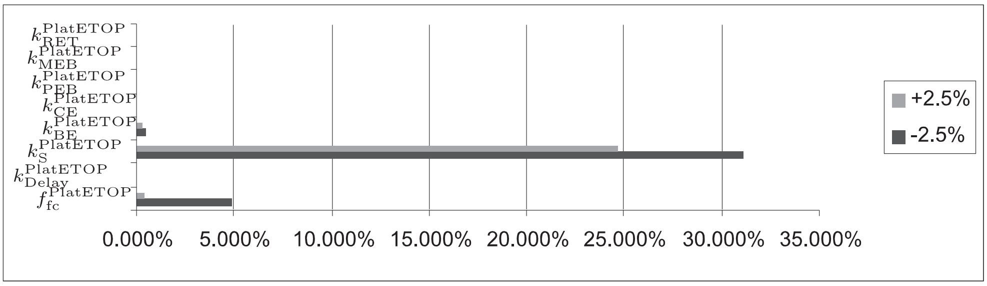

Figure 12. Sensitivity of toxicity parameters for the chemotherapy Platinum plus Etoposide

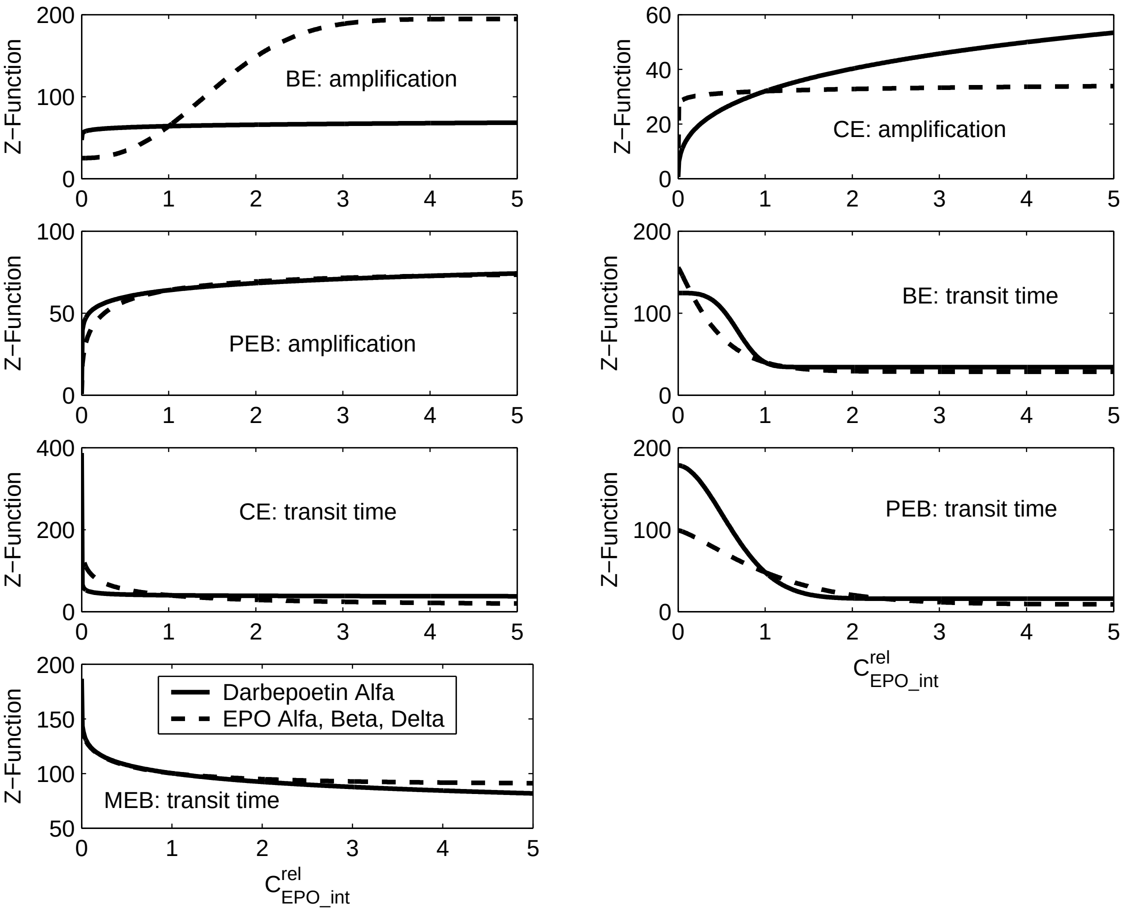

Figure 13. Z-functions of the amplifications in compartments BE, CE, PEB and the transition times in BE, CE, PEB, MEB

A.5 Simulation

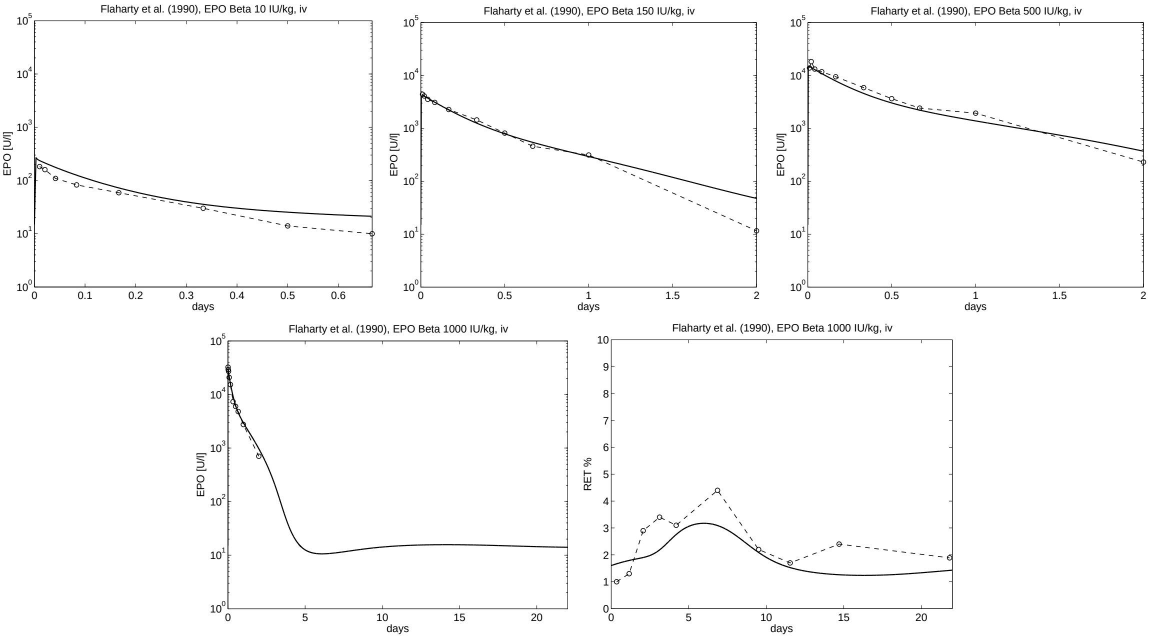

Figure 14. Serum concentration of erythropoietin, simulation (black line) and data (circle), data: [8]

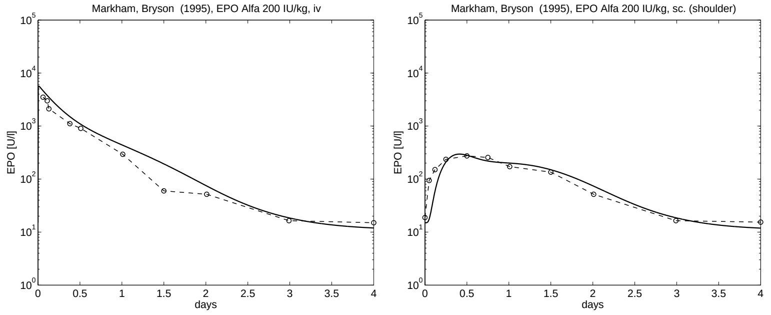

Figure 15. Serum concentration of erythropoietin (simulation and data), data: [9]

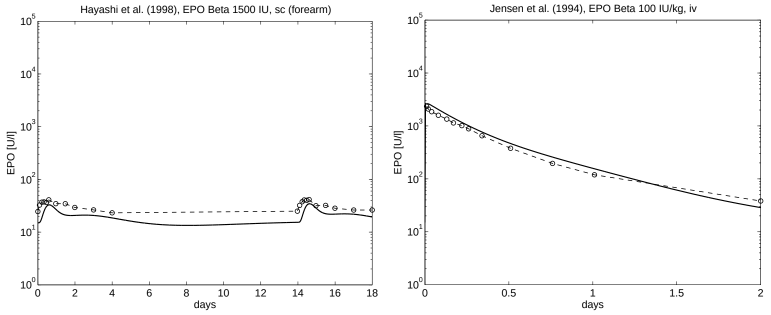

Figure 16. Serum concentration of erythropoietin (simulation and data), data: [10, 11]

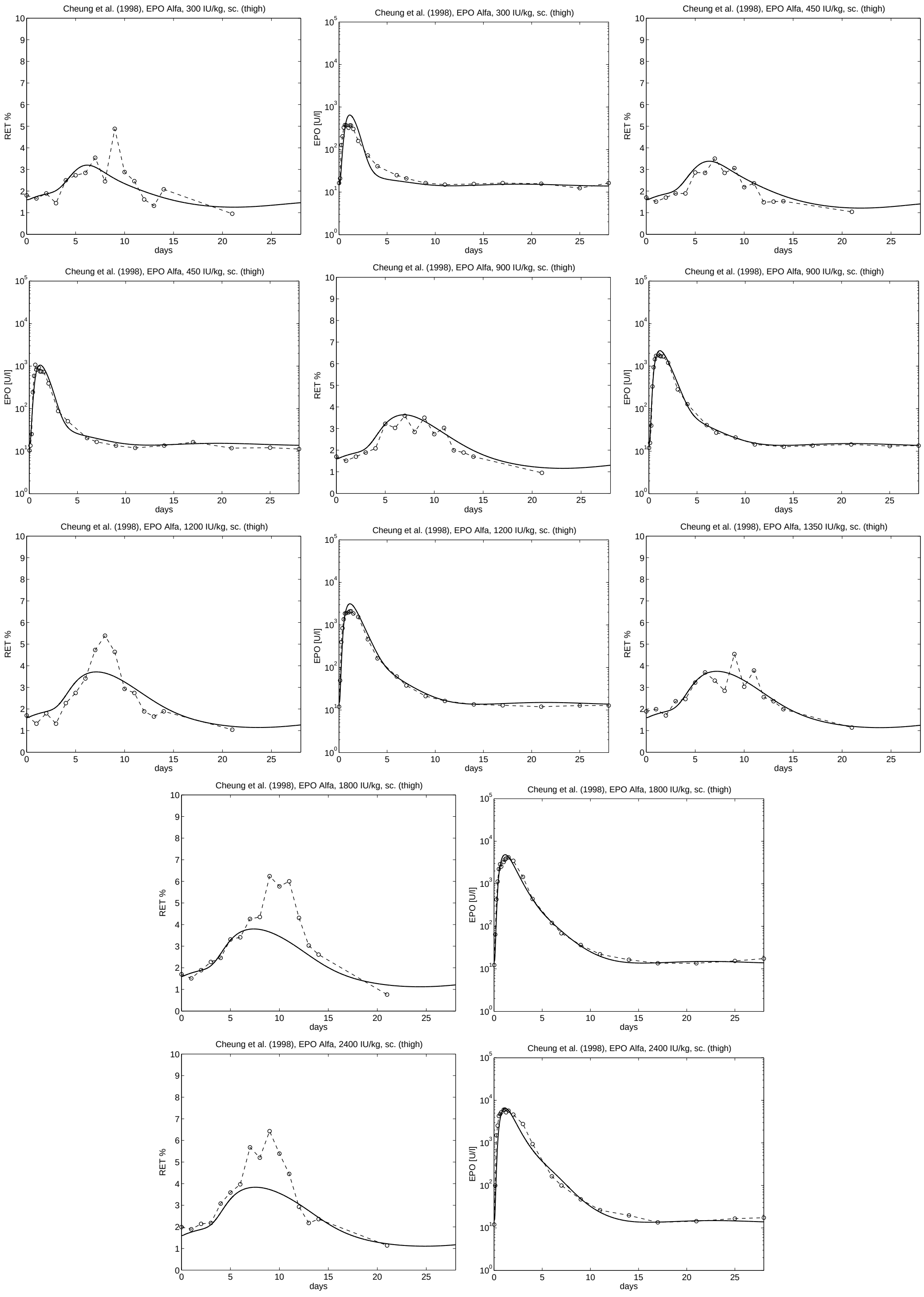

Figure 17. Serum concentration of erythropoietin and reticulocytes %, simulation (black line) and data (circle), data: [12]

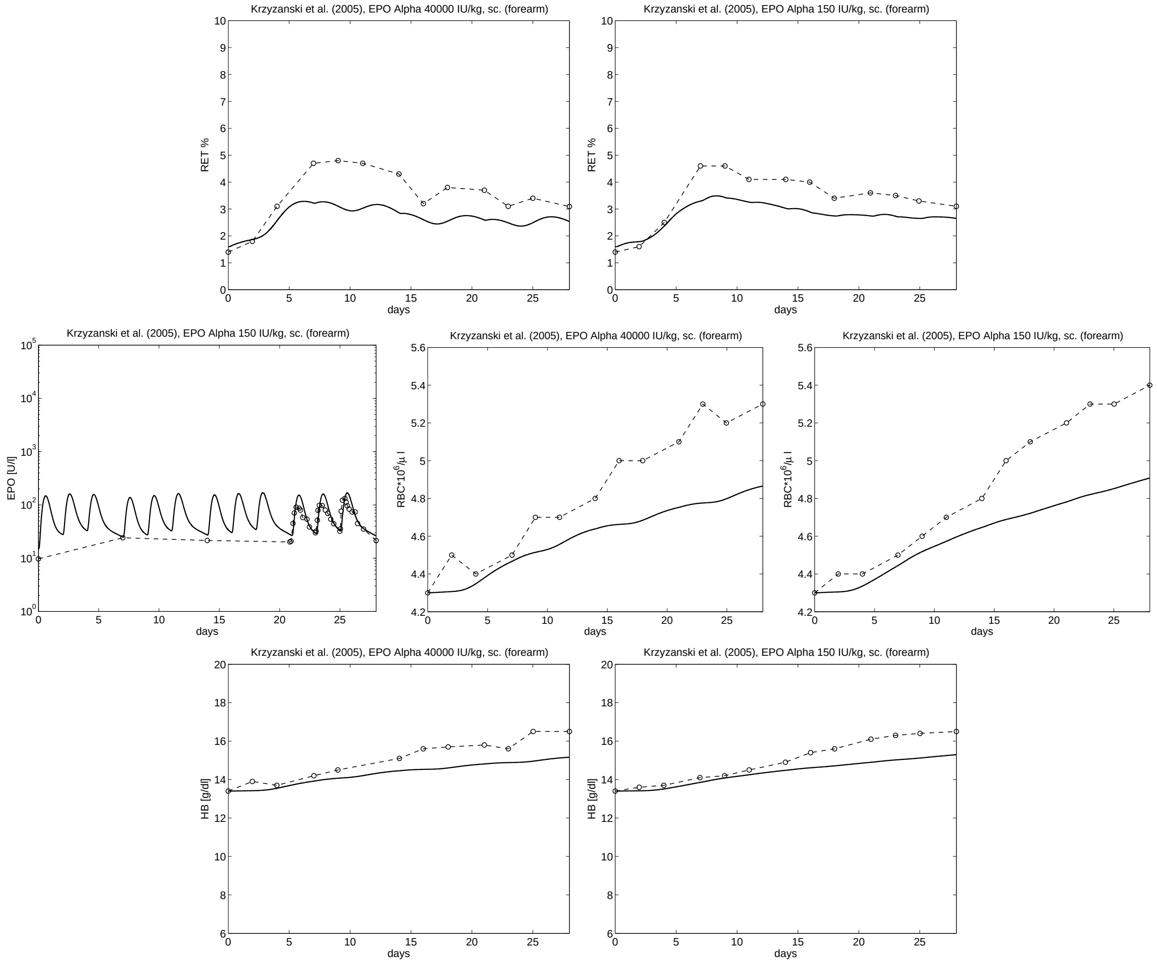

Figure 18. Serum concentration of erythropoietin, HB value, RBC, and reticulocytes %, simulation (black line) and data (circle), data: [7]

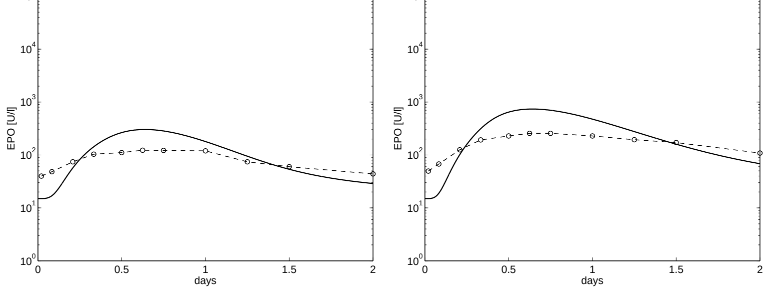

Figure 19. Serum concentration of erythropoietin, simulation (black line) and data (circle), data: [13]

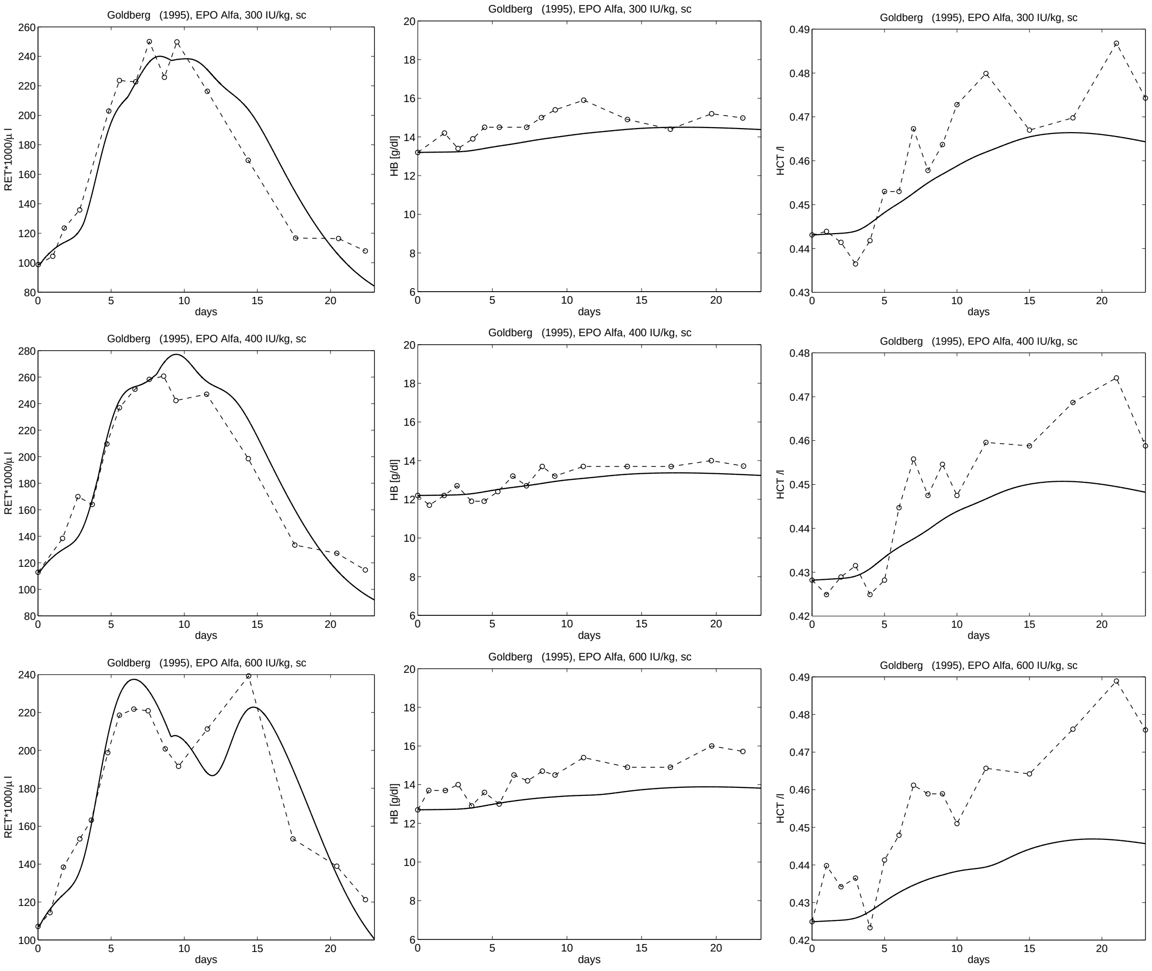

Figure 20. Serum concentration of erythropoietin, HB and HCT value, simulation (black line) and data (circle), data: [14], [15], only from subjects with baseline HCT of less than 48%. If the HCT rose above 55%, phlebotomy was performed.

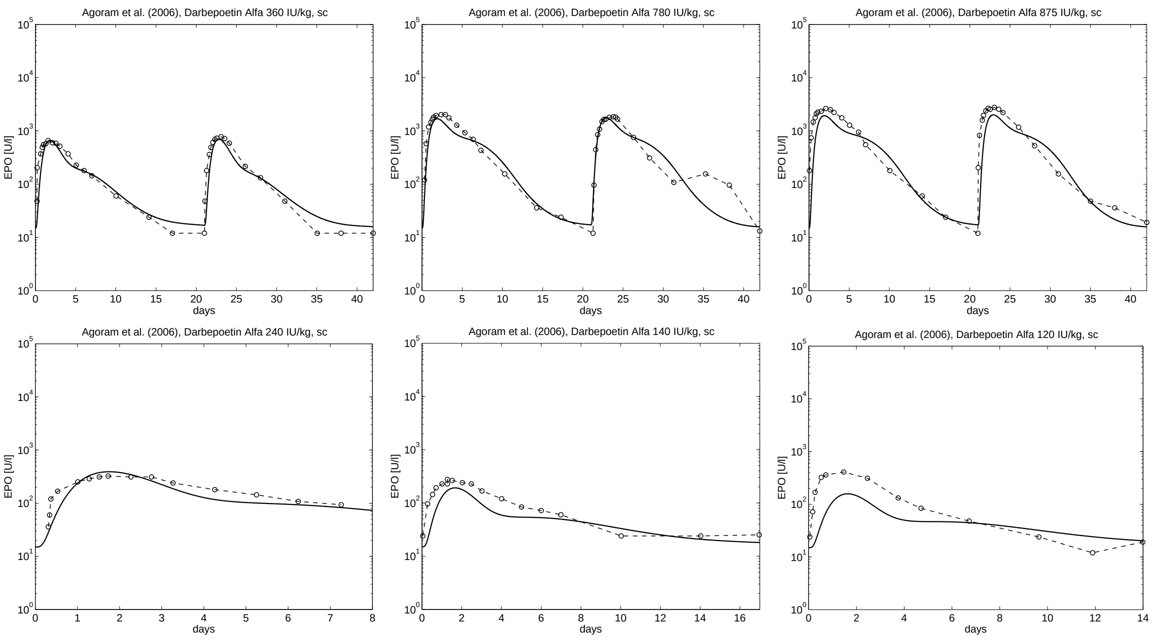

Figure 21. Serum concentration of erythropoietin, simulation (black line) and data (circle), data: [16]

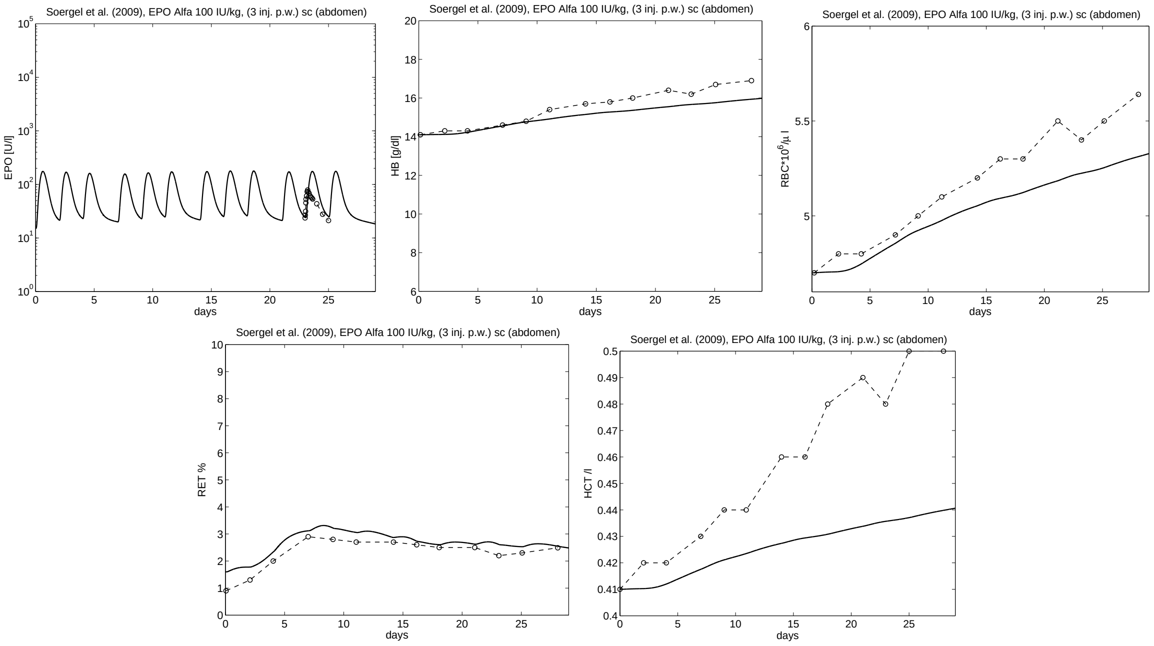

Figure 22. Serum concentration of erythropoietin, HB, RBC, reticulocytes % and HCT, simulation (black line) and data (circle), data: [17]

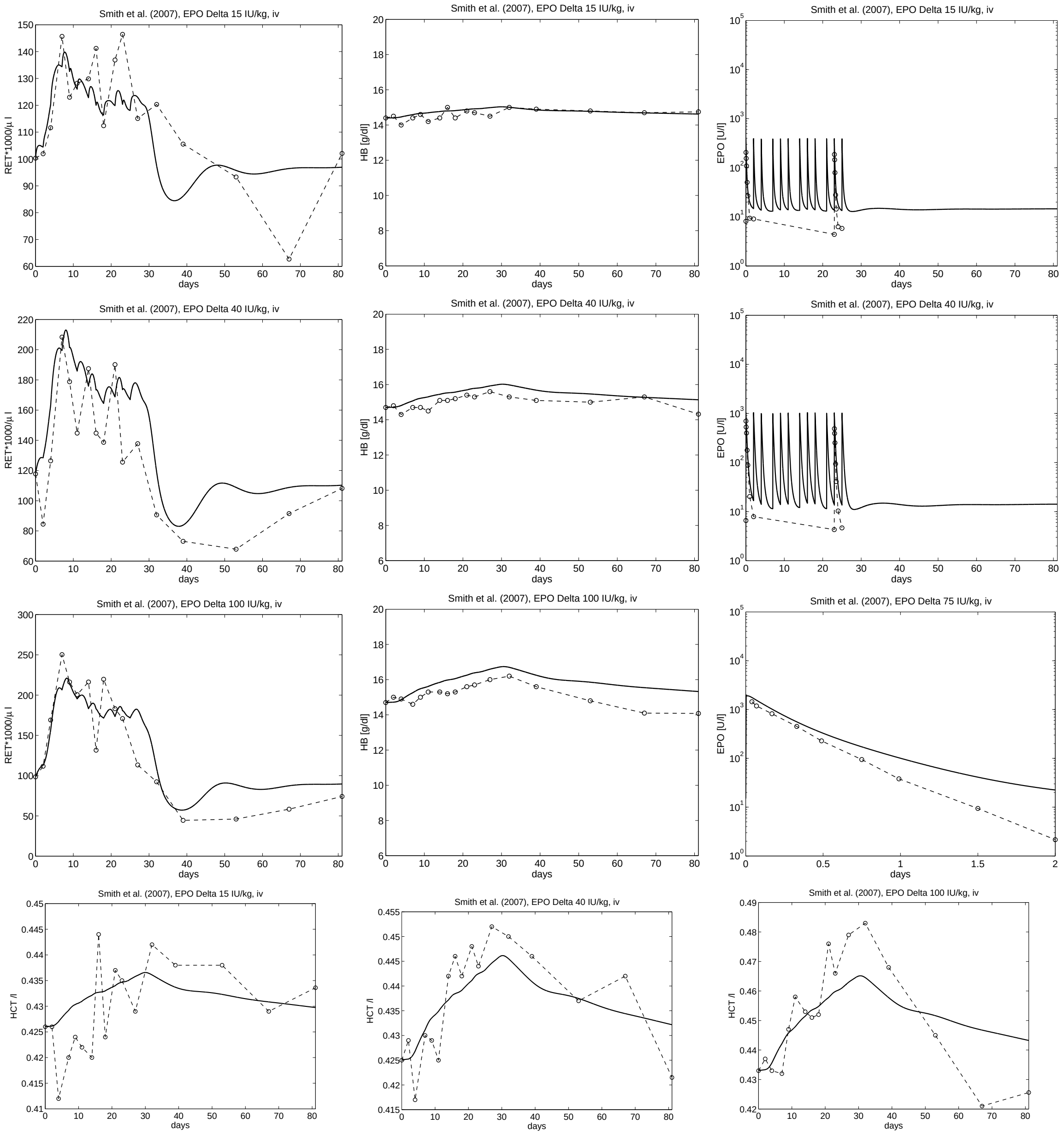

Figure 23. HB, reticulocytes %, Serum concentration of erythropoietin and HCT, simulation (black line) and data (circle), data: [18]

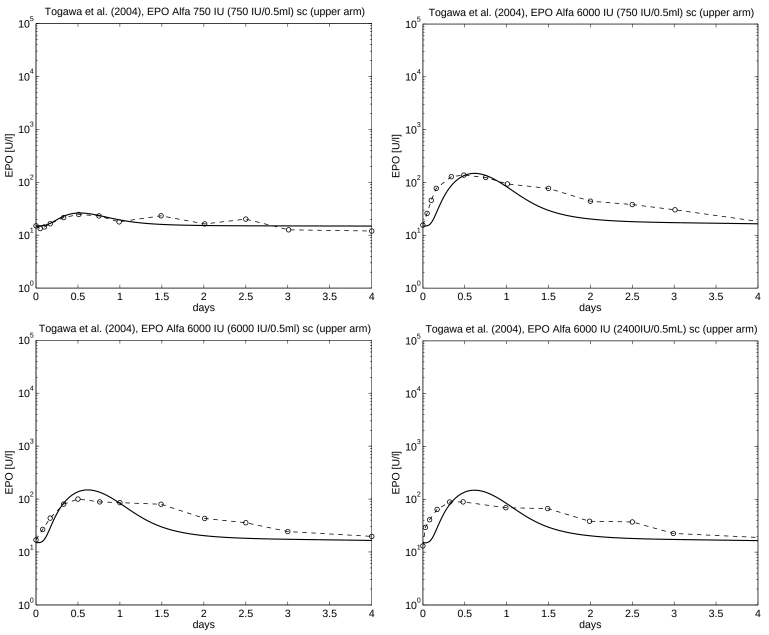

Figure 24. Serum concentration of erythropoietin, simulation (black line) and data (circle), data: [19]

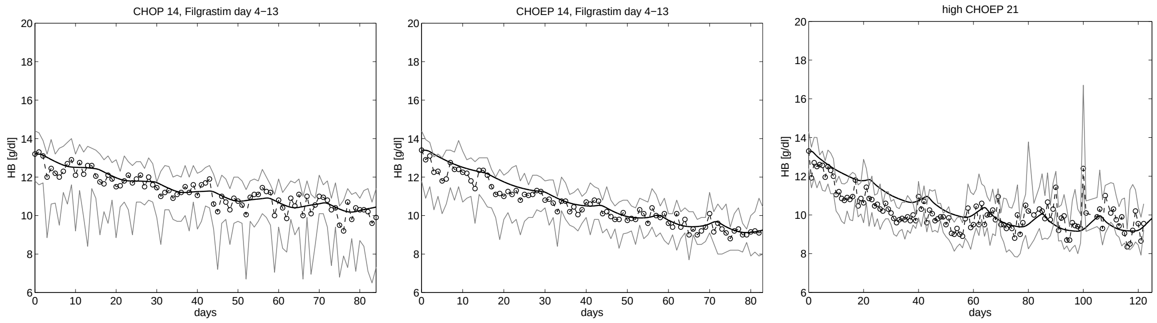

Figure 25. HB value simulation (black line) and data (circle), percentile 25, 75 (grey line), data: [20–22]

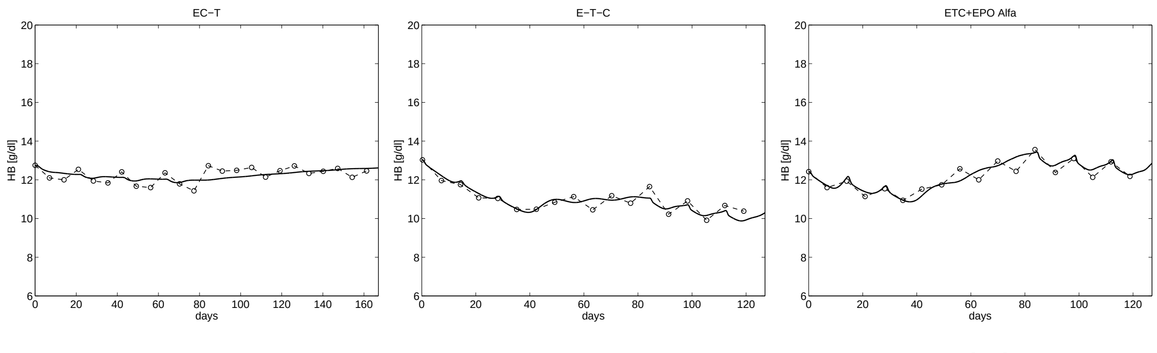

Figure 26. HB value, simulation (black line) and data (circle), data: [23]

References

1. Scholz M, Engel C, Loeffler M (2005) Modelling human granulopoiesis under polychemotherapy with g-csf support. J Math Biol 50: 397–439.

2. Wichmann H, Loeffler M (1985) Mathematical modeling of cell proliferation: Stem cell regulation in hemopoiesis, Vol. 1, 2. CRC Press.

3. Loeffler M, Pantel K, Wulff H, Wichmann H (1989) A mathematical model of erythropoiesis in mice and rats. part 1: Structure of the model. Cell Tissue Kinet 22: 13–30.

4. Pantel K (1987) Erweiterung eines kybernetischen Modelles der Erythropoese und dessen Anwendung für normale und pathologische Mäuse. Dissertation, Universität zu Köln (in German).

5. Wichmann H (1983) Computer modeling of erythropoiesis. In: Current Concepts in Erythropoiesis, Chapter V., John Wiley and Sons.

6. Franke M, Schmitz S (1993) Modell Mensch aus Übersicht über die Modelle zur Hämatopoese 1979-1993. Universität zu Köln.

7. Krzyzanski W, Wyska E (2008) Pharmacokinetics and pharmacodynamics of erythropoietin receptor in healthy volunteers. Naunyn-Schmiedeberg's Arch Pharmacol 377: 637–645.

8. Flaharty K, Caro J, Erslev A, Whalen J (1990) Pharmakokinetics and erythropoietic response to human recombinant erythropoietin in healthy men. Clin Pharmacol Ther 47: 557–564.

9. Markham A, Bryson H (1995) Epoetin alfa. a review of its pharmacodynamic and pharmacokinetic properties and therapeutic use in nonrenal applications. Drugs 49: 232–254.

10. Hayashi N, Kinoshita H, Yukawa E, Higuchi S (1998) Pharmacokinetic analysis of subcutaneous erythropoietin administration with nonlinear mixed effect model including endogenous production (7). Br J Clin Pharmacol 46: 11–19.

11. Jensen J, Madsen J, Jensen L, Pedersen E (1994) Reduction absorption and elimination of erythropoietin in uremia compared with healthy volunteers. J Am Soc Nephrol 5: 177–185.

12. Cheung W, Goon B, Guilfoyle M, Wachholtz M (1998) Pharmacokinetics and pharmacodynamics of recombinant human erythropoietin after single and multiple subcutaneous doses to healthy subjects. Clin Pharmacol Ther 8: 309–317.

13. McMahon F, Vargas R, Ryan M, Jain A, Abels R, et al. (1990) Pharmacokinetics and effects of recombinant human erythropoietin after intravenous and subcutaneous injections in healthy voluteers. Blood 76: 1718–1722.

14. Goldberg M (1995) Erythropoesis, erythropoetin, and iron metabolism in elective surgery: Preoperative strategies for avoiding allogeneic blood exposure. Am J Surg 170 : 37S–43S.

15. Rutherford C, Schneider T, Dempsey H, Kirn D, Brugnara C, et al. (1994) Efficacy of different dosing regimens for recombinant human erythropoietin in a simulated perisurgical setting: The importance of iron availability in optimizing response. Am J Med 96: 139–145.

16. Agoram B, Sutjandra L, Sullivan J (2006) Population pharmacokinetics of darbepoetin alfa in healthy subjects. Br J Clin Pharmacol 63: 41–52.

17. Sörgel F, Thyroff-Friesinger U, Vetter A, Vens-Cappell B, Kinzig M (2009) Bioequivalence of hx575 (recombinant human epoetin alfa) and a comparator epoetin alfa after multiple subcutaneous administrations. Pharmacology 38: 122–130.

18. Smith W, Dowell J, Pratt R (2007) Pharmacokinetics and pharmacodynamics of epoetin delta in two studies in healthy volunteers and two studies in patients with chronic kidney disease. Clinical Therapeutics 29: 1368–1380.

19. Togawa A, Tanaka T, Nagashima S, Keta H, Kobayashi Y, et al. (2004) A comparison of the bioequivalence of two formulations of epoetin alfa after subcutaneous injection. Br J Clin Pharmacol 58: 269–276.

20. Pfreundschuh M, Truemper L, Kloess M, Schmits R, Feller A, et al. (2004) 2-weekly or 3-weekly chop chemotherapy with or without etoposide for the treatment of young patients with good prognosis (normal ldh) aggressive lymphomas: results of the nhl-b1 trial of the dshnhl. Blood 104: 626–633.

21. Pfreundschuh M, Truemper L, Kloess M, Schmits R, Feller A, et al. (2004) 2-weekly or 3-weekly chop chemotherapy with or without etoposide for the treatment of elderly patients with aggressive lymphomas: results of the nhl-b2 trial of the dshnhl. Blood 104: 634–641.

22. Pfreundschuh M, Zwick C, Zeynalova S, Dührsen U, Pflüger KH, et al. (2008) Dose-escalated choep for the treatment of young patients with aggressive non-hodgkin's lymphoma: 2. results of the randomized high-choep trial of the german high-grade non-hodgkin's lymphoma study group (dshnhl). Ann Oncol 19: 545–552.

23. GBG. German breast group, available: http://www.germanbreastgroup.de /studien /adjuvant /gain/unterlagen.html accessed 2011 october 11.
